# Predictive modeling of heavy metal pollution and ecological risk for sustainable water quality management in the NY–NJ harbor system

**DOI:** 10.1007/s10661-026-15832-x

**Published:** 2026-08-31

**Authors:** Md Shahnul Islam, Sara Naveed, Huan Feng, Tapos Kumar Chakraborty

**Affiliations:** 1 Department of Earth and Environmental Studies, Montclair State University, Montclair, NJ 07043 USA; 2 Department of Environmental Science and Technology, Jashore University of Science and Technology, Jashore, 7408 Bangladesh

**Keywords:** Heavy metal pollution, Ecological risk modeling, Machine learning, Lower Passaic River, Environmental risk assessment

## Abstract

Evaluating and forecasting surface water quality is essential for protecting aquatic ecosystems and improving water resource management. This study introduces a novel paradigm that integrates machine learning (ML) with the potential ecological risk index (*PERI*) to dynamically forecast, rather than statically assess, ecological risks from heavy metal contamination in an urban estuarine environment. Surface water samples from the Lower Passaic River in New Jersey, USA, were analyzed for copper (Cu), lead (Pb), and mercury (Hg) across multiple sites and sampling campaigns. Concentrations ranged from 3.1 to 42.6 µg/L for Cu, 1.8 to 25.4 µg/L for Pb, and 0.12 to 1.36 µg/L for Hg, corresponding to *PERI* values spanning from 85.7 to 672.3, indicating moderate to very high ecological risk levels. Four ML algorithms, random forest (RF), support vector machine (SVM), extreme gradient boosting (XGBoost), and artificial neural network (ANN), were employed to model *PERI* based on pollution indices. According to the findings, model performance was ranked as ANN > RF > SVM > XGBoost. The ANN model demonstrated superior performance, achieving the lowest error (MAE = 2.25) with excellent predictive accuracy (a testing *R*^2^ of 0.981), and was identified as the most effective model. Variable importance analysis confirmed Hg as the dominant risk driver. This integrated approach demonstrates that coupling ML with ecological risk indices can effectively capture complex, nonlinear relationships between metal concentrations and ecological impact, significantly improving risk forecasting for dynamic urban rivers and providing a mechanistic understanding of the dominant risk drivers. The findings provide a transferable framework for identifying contamination hotspots, guiding remediation efforts, and supporting adaptive management.

**Graphical Abstract:**

**Figure:**
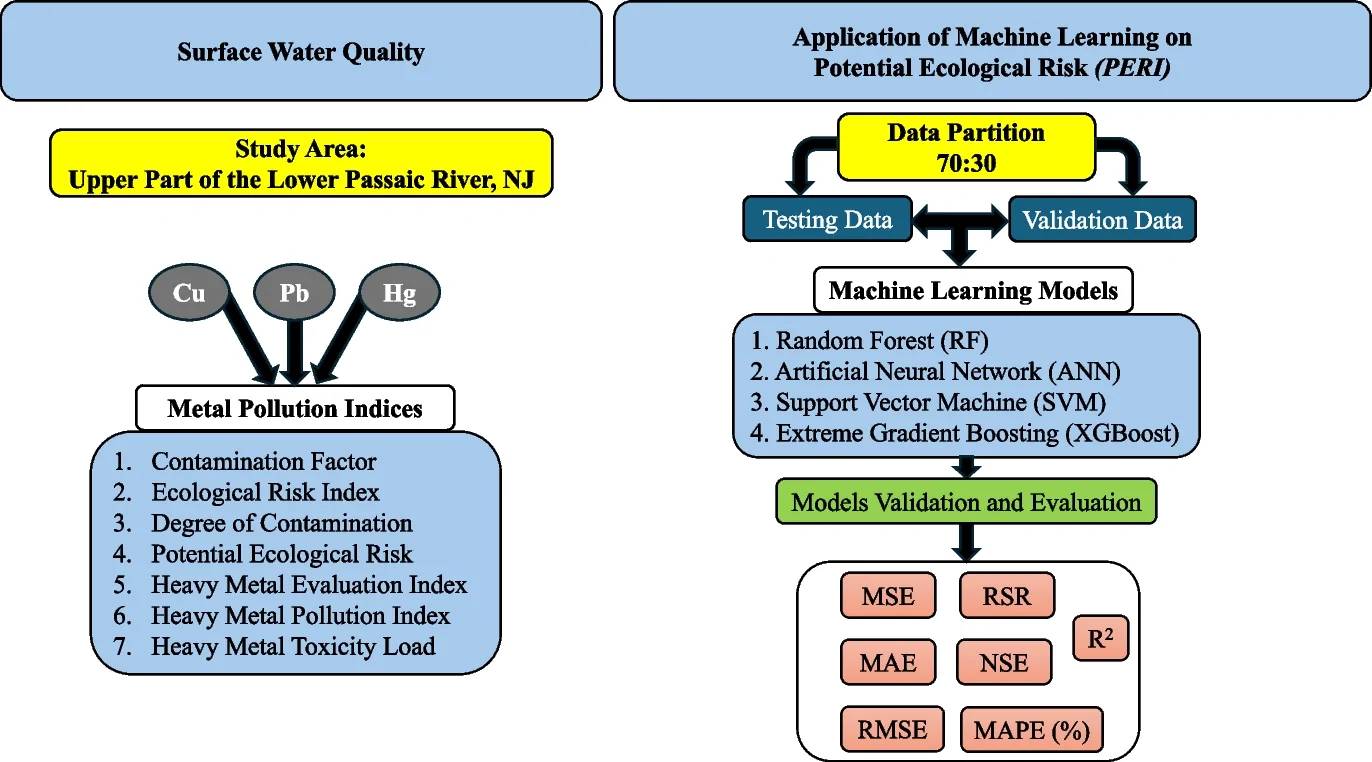

**Supplementary Information:**

The online version contains supplementary material available at https://doi.org/10.1007/s10661-026-15832-x.

## Highlights

• Cu, Pb, and Hg pollution assessed seasonally in the Lower Passaic River.

• Cu peaked in the fall; Pb and Hg showed no significant seasonal variation.

• ANN achieved the highest accuracy in predicting *PERI*.

• Hg dominated the *PERI* ecological risk.

• RM 8.4 and 10.2 were identified as hotspots requiring targeted remediation.

**Supplementary Information:**

The online version contains supplementary material available at https://doi.org/10.1007/s10661-026-15832-x.

## Introduction

Surface water systems are fundamental to ecological integrity, economic vitality, and public health. However, accelerated urbanization and industrial expansion have caused widespread degradation of water quality, particularly in densely populated estuarine environments (Chakraborty et al., 2022; Mansor et al., 2023; Riso et al., 1993; Sodré et al., 2012). Among the numerous contaminants, heavy metals such as Hg, Cu, and Pb are of exceptional concern because of their high environmental persistence, strong bioaccumulation potential, and pronounced ecological toxicity (Chakraborty et al., 2023; Lee et al., 2019; Nour & Garoub, 2026; Pandey & Kumari, 2023). These metals enter waterways primarily through industrial effluents, stormwater runoff, and municipal wastewater discharges, and can persist in sediments and the water column for decades, posing chronic risks to aquatic ecosystems and human populations (Acharjee et al., 2026; Mansor et al., 2023; Wiener & Suchanek, 2008).

The New York–New Jersey (NY–NJ) Harbor System epitomizes these challenges. Characterized by intense maritime commerce, a legacy of industrialization, and dense urban infrastructure, the estuary exhibits complex hydrodynamic regimes and pronounced environmental heterogeneity that govern contaminant fate and transport (Donovan et al., 2008; Iannuzzi & Ludwig, 2004; Islam et al., 2026; Wenning et al., 1994). Within this system, the Lower Passaic River (LPR) is emblematic of the broader conflict between historical contamination and contemporary restoration. As a tidal tributary directly influencing the larger harbor and a site listed on the U.S. EPA Superfund National Priorities List, the LPR has experienced decades of heavy metal loading while simultaneously being the focus of major dredging and remediation efforts (NOAA, 2007; USEPA, 2023, 2026). Consequently, the LPR and the greater NY–NJ Harbor represent an ideal system for evaluating data-driven approaches to pollution control and ecological restoration in highly urbanized estuarine environments.

Determining ecological risk in such an environment remains technically demanding. Conventional statistical and deterministic water quality models often rely on static assumptions and low-resolution datasets that fail to capture the nonlinear interactions and multivariate dependencies among environmental drivers (Abraj et al., 2022; Islam et al., 2025; Laarne et al., 2022). Consequently, these approaches exhibit limited predictive accuracy and poor generalizability when confronted with the pronounced spatial and temporal variability characteristic of tidal estuaries (Ahmed et al., 2024; Li et al., 2024). Risk assessments based solely on fixed-index calculations, such as the potential ecological risk index (*PERI*) provide valuable snapshots of contamination status but cannot anticipate how risk levels will evolve under changing environmental conditions.

Machine learning (ML) algorithms have emerged as transformative tools capable of overcoming these limitations by learning complex, nonlinear patterns from high-dimensional environmental data. Unlike traditional deterministic models, ML methods effectively handle the nonlinear relationships and complex interactions that characterize heavy metal pollution in dynamic systems (Bi et al., 2024; Wang et al., 2020) and support vector machines, in particular, can mitigate problems associated with small samples and local optima while maintaining high generalization performance (Vapnik, 1999). Recent applications of random forest (RF), support vector machine (SVM), artificial neural networks (ANN), and extreme gradient boosting (XGBoost) have demonstrated considerable success in predicting heavy metal concentrations (Hu et al., 2026; Wang et al., 2022; Yan & Yang, 2025) and classifying water quality status (Basirian et al., 2026; Xie et al., 2025). Recent review studies have consistently identified RF, SVM, ANN, and XGBoost as the most frequently applied and best-performing machine learning algorithms for water quality assessment and heavy metal prediction because of their ability to capture nonlinear relationships, robustness across diverse environmental datasets, and strong predictive performance (Essamlali et al., 2024; Mohan et al., 2025; Zhu et al., 2022). These models also represent complementary learning paradigms, including tree-based ensemble methods (RF and XGBoost), kernel-based learning (SVM), and deep nonlinear function approximation (ANN), enabling comprehensive comparison of different modeling strategies for ecological risk prediction. A small number of studies have begun to couple ML outputs with ecological risk indices such as *PERI* in an effort to move beyond simple concentration prediction (Bi et al., 2024; Fan et al., 2021). Despite these encouraging advances, most ML-based investigations focus narrowly on contaminant levels while neglecting the forecasting of integrated ecological risk metrics. Furthermore, no framework has yet systematically leveraged ML to predict spatiotemporally resolved *PERI* values while simultaneously quantifying the relative importance of the environmental drivers shaping ecological risk in complex urban estuaries.

To address this gap, the present study develops and validates a predictive framework that integrates *PERI* calculations with an ensemble of ML algorithms (RF, SVM, XGBoost, and ANN). This integration represents a paradigm shift from retrospective, static risk assessment toward prospective ecological risk forecasting. The framework not only enhances predictive accuracy but also provides interpretable insights into the key environmental factors governing heavy metal ecological risk, thereby strengthening the scientific basis for adaptive water quality management. The specific objectives of this study are to (i) analyze the variability of Hg, Cu, and Pb concentrations in the surface waters of the NY–NJ harbor system against established regulatory thresholds; (ii) apply ML-based variable importance methods to identify the dominant environmental predictors of ecological risk; and (iii) develop and rigorously validate a predictive model for *PERI* that supports dynamic risk assessment and improves the reliability and generalizability of ecological risk forecasts in heavily urbanized coastal ecosystems.

## Materials and methods

### Study area

The Lower Passaic River (LPR) is a tidal riverine–estuarine system in northeast New Jersey, extending approximately 17 miles from the Dundee Dam to Newark Bay. The LPR is divided into the upper and lower sections. The lower section is contaminated with hazardous substances, including polychlorinated biphenyls (PCBs), heavy metals, and dioxins, due to historical industrial activity (Iannuzzi & Ludwig, 2004; Olson & Tharp, 2020). This section of the river flows through urbanized areas, including Newark, Passaic, and Paterson, with chemical manufacturing, industrial waste disposal, and contamination stemming from combined sewer overflow (Islam et al., 2025).

The Lower Passaic River (LPR) receives freshwater inflow from the upstream Passaic River above the Dundee Dam, three main tributaries (the Saddle River, Third River, and Second River) as well as several smaller tributaries. Additional inputs include direct discharges from combined sewer overflows (CSOs), stormwater outfalls (SWOs), permitted municipal and industrial discharges, and surface runoff. Geologically, the LPR is a low-gradient, alluvial system with both tidal and nontidal flows (Donovan et al., 2008; Wallin et al., 2002). The bed is composed primarily of fine silt and clay, which serve as reservoirs for contaminants. These sediments are often disturbed by tidal action, storm events, and anthropogenic activities, leading to the redistribution of contaminants (Khairy et al., 2015).

### Data collection, analytical methods, and data analysis

Water quality data were sourced from the Passaic River Digital Database, a collaborative effort maintained by the United States Environmental Protection Agency (USEPA), the United States Army Corps of Engineers (USACE), the New Jersey Department of Environmental Protection (NJDEP), and the United States Fish and Wildlife Service (USFWS). The data were collected for a project called “Lower Passaic River Study Area, Current Conditions and Monitoring Programs.” The project was originally designed to guide the first “Year 1” of a multiyear investigative effort. However, due to various external factors, including the COVID-19 pandemic and site-specific flow conditions, “Year 1” activities and data were expanded over several calendar years (AECOM, 2022).

**Fig. 1:**
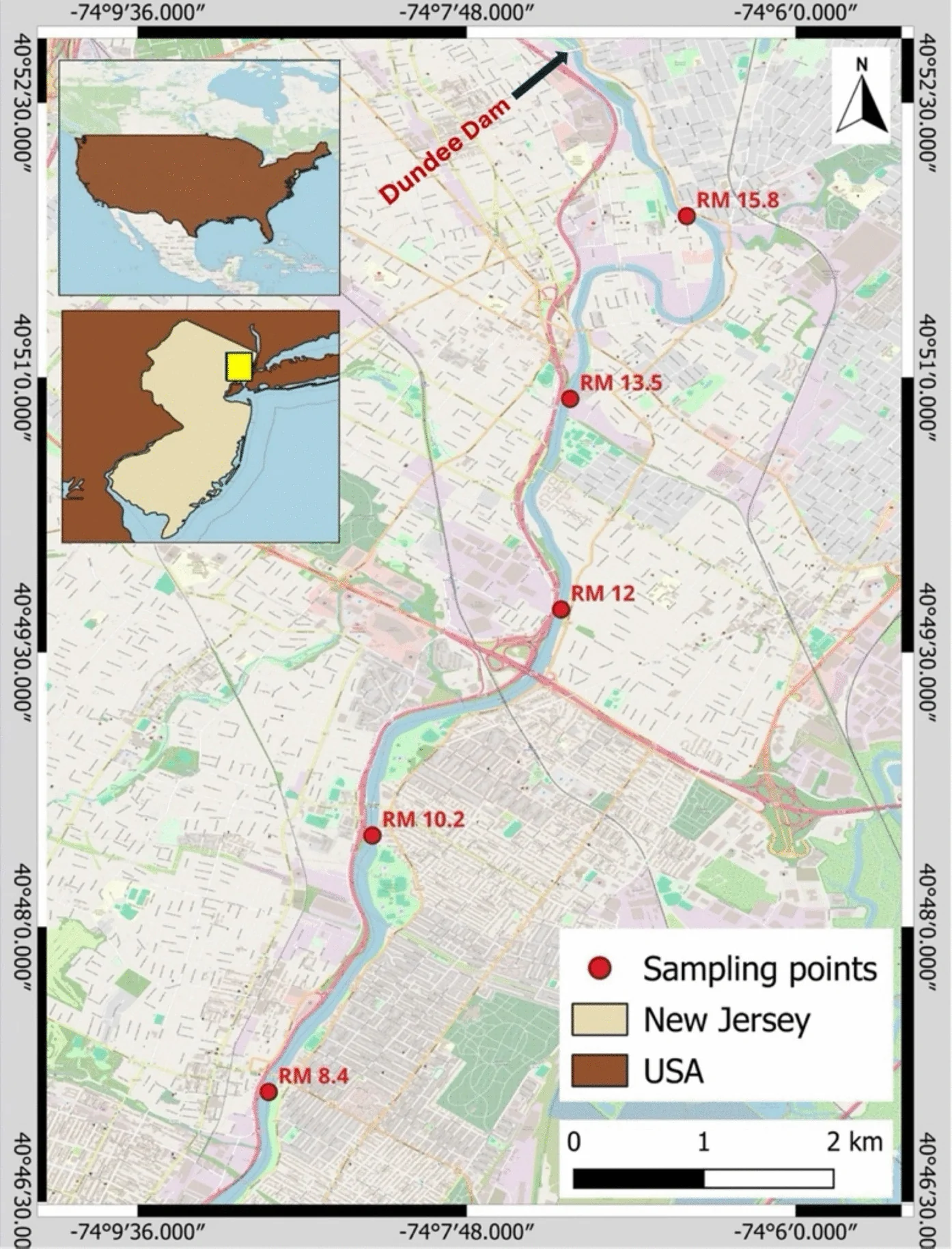
Location of the study area in the upper portion of the Lower Passaic River (LPR), extending from Dundee Dam to River Mile (RM) 8

Sampling was done on the upper part of the LPR, specifically between River Mile (RM) 8 and Dundee Dam. For this monitoring study, five sample sites, RM 8.4, RM 10.2, RM 12.0, RM 13.5, and RM 15.8, were chosen (Fig. 1). Water samples were collected during 11 sampling events conducted across three seasonal monitoring periods: December 2019 (fall), June to August 2020 (summer), and March to April 2021 (spring). During each sampling event, samples were generally collected at two tidal stages (flood and ebb). At RM 8.4, RM 10.2, RM 12.0, and RM 13.5, samples were collected at two depths (0.9 m [3 ft] below the surface and approximately 0.6 m [2 ft] above the river bottom), whereas RM 15.8 was sampled only at mid-depth because of site-specific conditions. This sampling design produced approximately 18 samples per event, resulting in a final dataset of 198 water samples. Field duplicate samples collected as part of the quality assurance/quality control (QA/QC) program were included in the final dataset. Samples were collected at 0.9 m (3 ft) below the water surface (AECOM, 2022). This study focused on three heavy metals, Cu, Pb, and Hg, based on their high toxicity, regulatory relevance, and documented occurrence in the Lower Passaic River system. These metals have been consistently reported as priority pollutants linked to historical industrial discharges and urban runoff in the region (AECOM, 2022; Iannuzzi et al., 2008).

Immediately after collection, all water samples were acidified to pH < 2 using ultrapure nitric acid and stored at 4 °C until laboratory analysis to prevent metal adsorption and ensure data integrity, following EPA Method 1669 (USEPA, 1996). Copper (Cu) and lead (Pb) concentrations were analyzed at ALS Environmental (Kelso, WA), while mercury (Hg) was analyzed at Brooks Applied Labs (Bothell, WA). EPA Method 6020 A (SW-846) was employed for Cu and Pb using inductively coupled plasma mass spectrometry (ICP-MS) with argon plasma. Mercury (Hg) analysis followed EPA Method 1631 (Revision E), using cold vapor atomic fluorescence spectrometry (CVAFS) (AECOM, 2022). Analytical QA/QC procedures followed EPA guidelines and included standard reference materials (SRMs), method blanks, and matrix spikes. Recovery rates for Cu, Pb, and Hg ranged from 92 to 106%, indicating high analytical accuracy. Method detection limits (MDLs) were 0.1 µg/L for Cu, 0.05 µg/L for Pb, and 0.002 µg/L for Hg. All measured values were within acceptable QA/QC limits as defined by USEPA (1996). Summary statistics, including detection frequencies and reporting limits (RLs) for heavy metals in water samples, are provided in Table S1. Physical–chemical parameters were measured using a YSI multiparameter sonde (EX02) (AECOM, 2022).

Microsoft Excel (v.16.85.2), JMP Pro (v.16.2.0), and RStudio (v.2024.04.1 + 748) were used for statistical analyses. Data were assessed for homogeneity and normality. Because the data did not satisfy the assumptions of normality, Spearman’s rank correlation was used to evaluate the relationships between heavy metals and the measured physicochemical parameters. Principal component analysis (PCA) with Varimax rotation was performed to identify the principal factors influencing relationships among physicochemical parameters and heavy metals in the LPR. Principal components with eigenvalues greater than 1 were retained according to the Kaiser criterion, and the rotated component loadings were used to facilitate interpretation of the variables contributing to each principal component.

### Pollution assessment indices

To comprehensively evaluate heavy metal pollution and ecological risk, this study employed both single-metal and integrated pollution indices. The contamination factor (*C*_*f*_) quantifies the contamination level of each individual metal relative to its background concentration, while the ecological risk index (*E*^*i*^_*r*_) incorporates both contamination and the toxic response of each metal to estimate its potential ecological risk. Integrated indices, including the contamination degree (*C*_*d*_), heavy metal evaluation index (*HEI*), heavy metal pollution index (*HPI*), heavy metal toxicity load (*HMTL*), and potential ecological risk index (*PERI*), provide an overall assessment of water quality by combining the effects of multiple heavy metals. The mathematical expressions, classification criteria, and references for these indices are presented in Table 1.

**Table 1:** Indices and classification criteria for heavy metal pollution and ecological risk assessment

Indices	Mathematical expression	Classification	Contamination degree	References
Contamination factor (*C*_*f*_)	${C}_{f}=\frac{{C}_{i}}{{C}_{b}}$ Here, *C*_*i*_ = measured concentration on the metal, *C*_*b*_ = background value. The background concentrations for river water are 1 µg/L for Cu, 0.2 µg/L for Pb, and 0.01 µg/L for Hg, respectively (Salomons & Förstner, 1984)	*C*_*f*_ < 1	Low	Hakanson (1980)
		1 ≤ *C*_*f*_ < 3	Moderate	
		1 ≤ *C*_*f*_ < 3	Considerable	
		*C*_*f*_ ≥ 6	Very high	
Ecological risk index (*E*^*i*^_*r*_)	${E}_{r}^{i}={T}_{r}^{i}\times {C}_{fi}$ Here, *T*^*i*^_*r*_ = toxic response factor of individual heavy metal. The *T*^*i*^_*r*_ values for Cu, Pb, and Hg are 5, 5, and 40, respectively	*E*^*i*^_*r*_ < 40	Low	Hakanson (1980)
		40 ≤ *E*^*i*^_*r*_ < 80	Moderate	
		80 ≤ *E*^*i*^_*r*_ < 160	High	
		160 ≤ *E*^*i*^_*r*_ < 320	Much higher	
		*E*^*i*^_*r*_ ≥ 320	Serious	
Contamination degree (*C*_*d*_)	$C_d=\sum\limits_{i=1}^nC_{fi}$	*C*_*d*_ < 8	Low	Hakanson (1980)
		8 ≤ *C*_*d*_ < 16	Moderate	
		16 ≤ *C*_*d*_ < 32	Considerable	
		*C*_*d*_ ≥ 32	Very high	
Potential ecological risk (*PERI*)	$PERI=\sum\limits_{i=1}^{n}{E}_{r}^{i}$	*PERI* < 150	Low-grade	Hakanson (1980)
		150 < *PERI* < 300	Moderate	
		300 < *PERI* < 600	Severe	
		*PERI* > 600	Serious	
Heavy metal evaluation index (*HEI*)	$HEI=\sum\limits_{i=1}^{n}\frac{{H}_{c}}{{H}_{mac}}$ Here, *H*_*c*_ represents the measured concentration, and *H*_*mac*_ denotes the maximum allowable concentration for the *i*^*th*^ parameter. The *H*_*mac*_ values for Cu, Pb, and Hg are 1300 µg/L, 10 µg/L, and 2 µg/L, respectively (USEPA, 2024)	*HEI* < 400	Low	Proshad et al. (2021)
		400 ≤ *HEI* < 800	Moderate	
		*HEI* ≥ 800	High	
Heavy metal pollution index (*HPI*)	$HPI=\sum\limits_{i=1}^{n}\frac{{W}_{i}{Q}_{i}}{{W}_{i}}$ Here, *W*_*i*_ represents the unit weight of the *i*^*th*^ heavy metal, and *Q*_*i*_ is the subindex for the *i*^*th*^ HM; *Q*_*i*_ is calculated as follows: ${Q}_{i}=\sum\limits_{i=1}^{n}\frac{\left\{\left.{C}_{i}(-){I}_{i}\right\}\right.}{{S}_{i}-{I}_{i}}\times 100$ Here, *S*_*i*_ and *I*_*i*_ represent the standard and ideal values for drinking water, respectively, as specified by USEPA (2024) for heavy metals in µg/L	*HEI* < 100	Low	Saha and Paul (2019)
		*HEI* = 100	Threshold risk	
		*HEI* > 100	High	
Heavy metal toxicity load (*HMTL*)	$HMTL=\sum\limits_{i=1}^{n}{C}_{i}-HIS$ Here, *HIS* represents the hazard intensity score. *HIS* is derived from the Agency for Toxic Substances and Disease Registry (ATSDR), considering the frequency of these toxic substances appearing on the National Priorities List (NPL), their toxicity levels, and the potential for human exposure. Table 5 (toxicity of heavy metals (µg/L) and heavy metal toxicity load (µg/L)) lists the highest allowable thresholds for determining the *HIS* that have been approved by ATSDR (2025)			Saha and Paul (2019)

Among these indexes, *PERI* was selected as the target variable for the machine learning models because it integrates both the contamination level and the toxic response of individual heavy metals into a single ecological risk metric. Compared with the other indexes, *PERI* provides a more comprehensive representation of ecological risk and is widely used for environmental risk assessment. The machine learning framework was therefore developed to predict *PERI* from the measured environmental variables, allowing rapid estimation of ecological risk without requiring direct calculation of the index from all heavy metal measurements.

### Machine learning methods

**Fig. 2:**
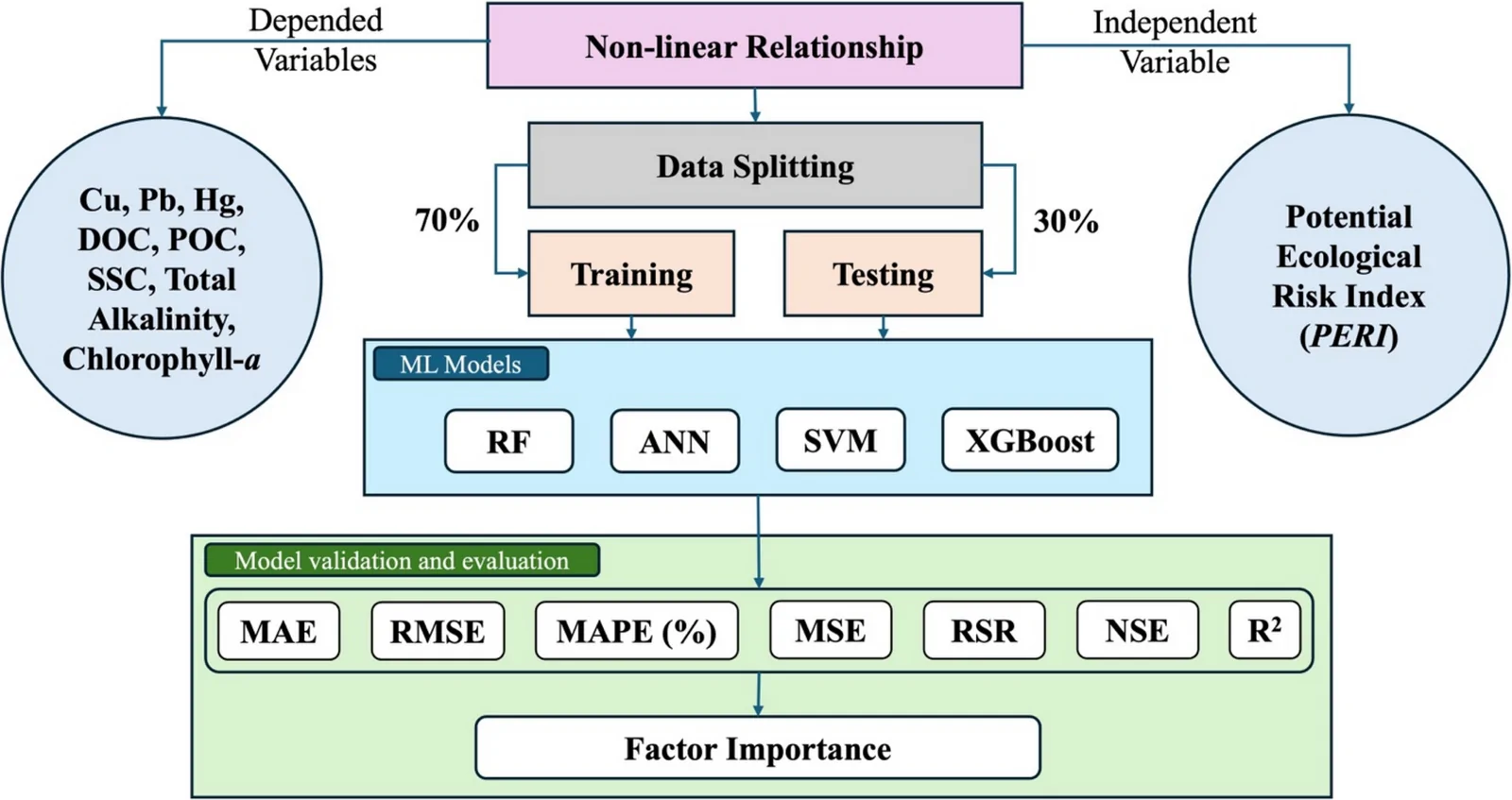
Workflow of the proposed ML modeling approach for *PERI* prediction

We assessed the performance of the four machine learning models: random forest (RF), support vector machine (SVM), extreme gradient boosting (XGBoost), and artificial neural network (ANN), as detailed in Table S2. RF, an ensemble learning method, generates several decisions during training and produces the average prediction of individual decision trees, effectively reducing overfitting and improving accuracy (Breiman, 2001). SVM is a supervised learning algorithm that constructs an optimal hyperplane in a high-dimensional space to separate data classes and can be adapted for regression functions using kernel functions (Cortes & Vapnik, 1996). XGBoost, a scalable implementation of gradient boosting, enhances model performance through regularization techniques and efficient handling of missing data (Chen & Guestrin, 2016). ANN, inspired by the structure of the biological neural networks, contains interconnected layers of nodes (neurons) that learn hierarchical feature representation through iterarive weight optimization using backpropagation (Rumelhart et al., 1986). In this study, a 70:30 ratio was employed to split the dataset into the training and testing sets, respectively, during the application of machine learning techniques. The detailed procedures for data preprocessing, dataset partitioning, and the hyperparameter tuning process for each model are described in Sects.“Data preprocessing and partitioning” and “Model development and hyperparameter optimization,” respectively. The overall workflow of the proposed ML modeling methodology is illustrated in Fig. 2.

#### Data preprocessing and partitioning

The machine learning models were developed to predict the potential ecological risk index (*PERI*). The predictor variables consisted of chlorophyll-*a*, total alkalinity, dissolved organic carbon (DOC), particulate organic carbon (POC), suspended sediment concentration (SSC), and the concentrations of Cu, Pb, and Hg. Prior to model development, the dataset was examined for missing values, and no imputation was required because all variables were complete. Heavy metal concentrations and physicochemical parameters were screened for extreme values; however, no observations were removed or modified.

Feature scaling was applied only to algorithms requiring normalized inputs. Specifically, the ANN model employed min–max normalization, with normalization parameters estimated exclusively from the training dataset and subsequently applied unchanged to the cross-validation and testing datasets to prevent data leakage. The remaining algorithms were implemented according to the requirements of their respective packages and did not require feature scaling.

The dataset was randomly partitioned into training (70%) and testing (30%) subsets using the “createDataPartition()” function with a fixed random seed (“*set.seed*(1234)”). The same training and testing subsets were used for all machine learning models to ensure a fair comparison of predictive performance. Hyperparameter tuning and model selection were conducted exclusively on the training dataset using fivefold cross-validation. During each iteration, fourfolds were used for model training and onefold was used for validation, with each fold serving once as the validation set. After the optimal hyperparameters were identified, each model was retrained using the complete training dataset and subsequently evaluated on the independent testing dataset. The testing dataset was not used during model development or hyperparameter optimization, thereby preventing data leakage and ensuring an unbiased assessment of predictive performance. Because the dataset did not exhibit strong spatial or temporal dependence, random partitioning was considered appropriate for model development and evaluation.

#### Model development and hyperparameter optimization

Model development included four supervised learning algorithms: random forest (RF), support vector machine (SVM), extreme gradient boosting (XGBoost), and artificial neural network (ANN). All analyses were conducted in the RStudio environment. The common model development workflow, including data partitioning, cross-validation, and prevention of data leakage, is described in Sect. “Data Preprocessing and Partitioning.” The algorithm-specific implementation and hyperparameter optimization procedures are described below.

- Random forest (RF): the random forest model was implemented using the “randomForest” package. The optimal number of variables randomly selected at each split (“*mtry*”) was determined through systematic hyperparameter optimization using fivefold cross-validation. Candidate “*mtry*” values ranging from 2 to 8 were evaluated, corresponding to the eight predictor variables included in the model. The optimal value (“*mtry*” = 6) was selected based on the lowest average cross-validated root mean square error (RMSE). The final model was trained using 500 trees (“*ntree*” = 500) with the selected “*mtry*” value.
- Support vector machine (SVM): The SVM model was implemented using the “caret” package with a radial basis function (RBF) kernel. Candidate combinations of the kernel parameters (“*sigma*” and “C”) were evaluated using fivefold cross-validation, and the combination with the lowest average cross-validated RMSE was selected. The final model was trained using the optimal hyperparameter combination.
- Extreme gradient boosting (XGBoost): the XGBoost regression model was implemented using the “xgboost” package. Model hyperparameters, including the learning rate (*eta* = 0.30), maximum tree depth (*max_depth* = 6), subsample ratio (*subsample* = 0.80), and column sampling ratio (*colsample_bytree* = 0.80), were selected based on preliminary experiments to achieve stable predictive performance while minimizing overfitting. A maximum of 100 boosting rounds was specified, and the optimal number of boosting rounds was determined using fivefold cross-validation with early stopping. The final model was trained using the selected hyperparameters and the optimal number of boosting rounds
- Artificial neural network (ANN): the ANN model was developed using the “neuralnet” package. Several candidate network architectures were evaluated using fivefold cross-validation. The selected architecture consisted of two hidden layers containing five and three neurons, respectively, as it provided stable convergence and consistently good predictive performance while maintaining a relatively simple network structure. The model employed the hyperbolic tangent activation function (*tanh*), the resilient backpropagation (*rprop* +) learning algorithm, and a maximum of 1,000,000 training steps to ensure convergence. The final model was trained using the selected architecture and hyperparameters, and model predictions were subsequently transformed back to the original *PERI* scale for performance evaluation.

### Accuracy assessment of ML models

The evaluation of the ML models was performed using the coefficient of determination (*R*^2^), mean absolute error (MAE), mean absolute percentage error (MAPE), and root mean square error (RMSE) (de Myttenaere et al., 2016). The most commonly used metric for assessing regression models is R^2^. Higher numbers on a scale of 0 to 1 suggest a better match, while lower values show a worse fit. The Nash–Sutcliffe Efficiency (NSE) is widely used in hydrology and water quality modeling; it ranges from − ∞ to 1, with values closer to 1 indicating better performance (values ≤ 0 indicate the model is no better than using the observed mean). The average relative error between estimated and measured values is measured by RMSE. The mean absolute value of the error between the measured and anticipated values is known as the MAE. Because of its natural interpretation in terms of relative error, MAPE, which focuses on percentage error, is frequently utilized in practice (Liang et al., 2025). The mathematical formulations of these performance metrics are presented in Eqs. 1, 2, 3, 4, and 5.

1 $${R}^{2}=1-\frac{\sum_{i=1}^{n}{({y}_{i}-{\widehat{y}}_{i})}^{2}}{\sum_{i=1}^{n}{\left({y}_{i}-\overline{y }\right)}^{2}}$$

2 $$NSE=1-\left[\frac{\sum_{i=1}^{n}{({y}_{i}-{\widehat{y}}_{i})}^{2}}{\sum_{i=1}^{n}{\left({y}_{i}-\overline{y }\right)}^{2}}\right]$$

3 $$MAE=\frac{1}{n}{\sum}_{i=1}^{n}\left|{y}_{i}-{\widehat{y}}_{i}\right|$$

4 $$MAPE=\frac{1}{n}{\sum}_{i=1}^{n}\left|\frac{{y}_{i}-{\widehat{y}}_{i}}{{y}_{i}}\right|\times 100\%$$

5 $$RMSE=\sqrt{\frac{1}{n}\times {\sum}_{i=1}^{n}{\left({y}_{i}-{\widehat{y}}_{i}\right)}^{2}}$$

where *y*_*i*_ denotes the measured value, *ŷ*_*i*_ signifies the estimated value, *n* represents the number of samples, and *ȳ*_*i*_ indicates the mean value of the measured data.

## Results

### Statistical analysis of heavy metal concentrations

The seasonal distribution of Cu, Pb, and Hg is highlighted in Table 2 at five sample locations along the LPR during the fall, spring, and summer. The data show distinct seasonal and spatial patterns, which vary in average concentrations across different locations. RM 8.4 recorded the highest values for Cu and Pb in most seasons. For Hg, while concentrations were relatively low, subtle seasonal differences were observed.

**Table 2:** Seasonal and spatial variation in the concentrations (µg/L) of trace metals (Cu, Pb, and Hg) in surface water across locations in the Lower Passaic River (LPR), with comparison to background values and regulatory limits

Season	Location		Cu	Pb	Hg
Fall	RM 8.4	Mean ± SD	3.434 ± 1.603	1.566 ± 1.844	0.0184 ± 0.031
		Range	2.08 − 7.61	0.162 − 6.28	0.0013 − 0.122
	RM 10.2	Mean ± SD	3.177 ± 0.859	1.208 ± 1.45	0.011 ± 0.013
		Range	2.1 − 5.43	0.28 − 4.27	0.0012 − 0.0411
	RM 12	Mean ± SD	2.688 ± 0.3853	0.4427 ± 0.2996	0.0027 ± 0.0027
		Range	1.85 − 3.25	0.152 − 1.11	0.0008 − 0.0097
	RM 13.5	Mean ± SD	2.7293 ± 0.4897	0.4842 ± 0.516	0.0026 ± 0.0037
		Range	1.87 − 3.78	0.136 − 1.7	0.0007 − 0.0125
	RM15.8	Mean ± SD	2.394 ± 0.227	0.2124 ± 0.1087	0.0014 ± 0.001
		Range	2.05 − 2.74	0.114 − 0.456	0.0006 − 0.0036
Spring	RM 8.4	Mean ± SD	2.11 ± 0.0572	0.4568 ± 0.0996	0.0041 ± 0.0011
		Range	2.05 − 2.18	0.347 − 0.547	0.0027 − 0.0052
	RM 10.2	Mean ± SD	1.872 ± 0.1256	0.2608 ± 0.0481	0.0019 ± 0.0003
		Range	1.74 − 2.03	0.22 − 0.338	0.0017 − 0.0023
	RM 12	Mean ± SD	2.1175 ± 0.0754	0.3008 ± 0.0378	0.0021 ± 0.0007
		Range	2.01 − 2.17	0.25 − 0.335	0.0012 − 0.0028
	RM 13.5	Mean ± SD	2.185 ± 0.1363	0.4108 ± 0.0641	0.0027 ± 0.0008
		Range	2.05 − 2.35	0.319 − 0.461	0.0019 − 0.0037
	RM15.8	Mean ± SD	2.325 ± 0.3041	0.389 ± 0.113	0.0025 ± 0.0004
		Range	2.11 − 2.54	0.309 − 0.469	0.0022 − 0.0028
Summer	RM 8.4	Mean ± SD	3.7367 ± 1.4555	1.6063 ± 2.0615	0.0077 ± 0.0071
		Range	2.61 − 5.38	0.121 − 3.96	0.0008 − 0.0151
	RM 10.2	Mean ± SD	2.82 ± 0.0781	0.4077 ± 0.4312	0.0036 ± 0.003
		Range	2.77 − 2.91	0.103 − 0.901	0.0008 − 0.0068
	RM 12	Mean ± SD	2.965 ± 0.3082	0.6328 ± 0.3803	0.0027 ± 0.0014
		Range	2.66 − 3.25	0.211 − 1.12	0.0009 − 0.0042
	RM 13.5	Mean ± SD	2.52 ± 0.07	0.1993 ± 0.0178	0.0008 ± 0.0002
		Range	2.47 − 2.6	0.179 − 0.212	0.0007 − 0.001
	RM15.8	Mean ± SD	2.865 ± 0.46	0.239 ± 0.1004	0.0009 ± 0.0005
		Range	2.54 − 3.19	0.168 − 0.31	0.0006 − 0.0013
Background value			1^a^	0.2^a^	0.01^a^
Permissible limit—aquatic life			−	65 (acute)^b^	1.4 (acute)^b^
			−	2.5 (chronic)^b^	0.77 (chronic)^b^
Permissible limit—drinking water			1300^c^	10^c^	2^c^
Toxicity reference value (TRVs)			9^d^	2.5^d^	0.0028^d^

^a^ Salomons and Förstner (1984)

^b^ USEPA (1986)

^c^ USEPA (2024)

^d^ USEPA (1999)

**Fig. 3:**
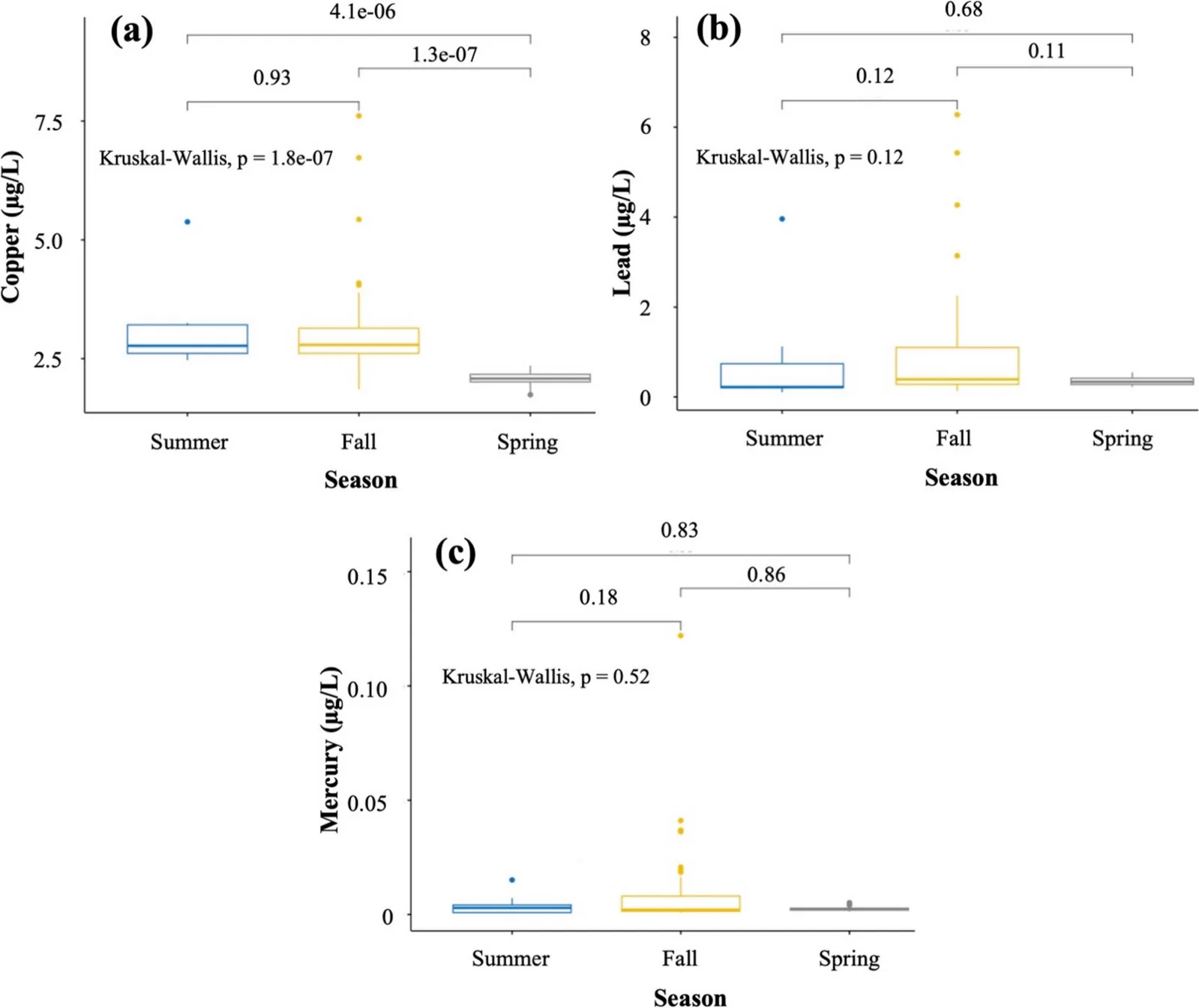
Seasonal variation of trace metal concentrations of **a** copper (Cu), **b** lead (Pb), and **c** mercury (Hg) in surface water samples collected from the upper portion of the Lower Passaic River (LPR). Box plots represent data distributions across summer, fall, and spring seasons. Kruskal–Wallis test results indicate a statistically significant seasonal difference for Cu, Pb, and Hg. Pairwise comparisons are labeled with corresponding *p*-values

The seasonal variation of heavy metals (Cu, Pb, and Hg) is depicted in Fig. 3, with box plots representing their concentrations during summer, fall, and spring. Copper exhibited statistically significant seasonal differences (Kruskal–Wallis, *p* = 1.8E-07), with concentrations highest in fall and lowest in spring (Fig. 3a). In pairwise comparisons, summer versus fall showed no significant difference (*p* = 0.93), summer versus spring was highly significant (*p* = 4.1E-06), and fall versus spring was the most significant (*p* = 1.3E-07). In contrast, Pb and Hg did not show any significant seasonal variation (Kruskal–Wallis, *p* = 0.12 for Pb and *p* = 0.52 for Hg).

### Variation of physicochemical parameters

Various sample locations (RM 8.4, RM 10.2, RM 12, RM 13.5, and RM 15.8) and seasons (fall, spring, and summer) exhibited spatial and seasonal variations in the physical and chemical parameters of the LPR. Chlorophyll-*a* concentration demonstrated significant seasonal variations, along with the highest levels observed during summer (Table 3). The total alkalinity showed noticeable fluctuations, with a decline relative to spring and summer levels. The dissolved organic carbon (DOC) and particulate organic carbon (POC) concentrations showed minor spatial and seasonal differences, with relatively stable values across the sampling locations and seasons (Table 3). The suspended sediment concentration (SSC) was highest in summer.

**Table 3:** Seasonal variation in physicochemical parameters (mean ± SD) across locations in the Lower Passaic River (LPR), compared with aquatic life criteria

Parameters	Location	Seasons			Aquatic life criteria
		Fall	Spring	Summer	
Chlorophyl-*a* (mg/m^3^)	RM 8.4	11.02 ± 10.42	10.48 ± 3.08	63.1 ± 41.71	20^a^
	RM 10.2	11.96 ± 11.4	9.56 ± 1.16	39.43 ± 29.68	
	RM 12	4.7 ± 3.03	15.13 ± 2.39	3.7 ± 1.89	
	RM 13.5	4.13 ± 3.17	15.38 ± 3.67	1.7 ± 0.7	
	RM 15.8	3.54 ± 1.63	13.6 ± 1.56	0.95 ± 0.21	
Total alkalinity (As CaCO_3_) (mg/L)	RM 8.4	84.53 ± 16.35	58.75 ± 3.2	88 ± 14.73	20^b^
	RM 10.2	86.67 ± 15.34	57.4 ± 2.88	88 ± 13.86	
	RM 12	84.81 ± 16.66	56 ± 0	82.5 ± 13.28	
	RM 13.5	87 ± 18.69	56.5 ± 0.58	86 ± 13.08	
	RM 15.8	85.25 ± 21.36	51 ± 2.83	75 ± 8.49	
DOC (mg/L)	RM 8.4	3.43 ± 0.71	3.93 ± 0.44	4.02 ± 0.35	8.3
	RM 10.2	3.45 ± 0.53	3.94 ± 0.46	4.09 ± 0.19	
	RM 12	3.59 ± 0.4	4.15 ± 0.51	4.32 ± 0.27	
	RM 13.5	3.47 ± 0.44	4.05 ± 0.66	4.15 ± 0.26	
	RM 15.8	3.35 ± 0.53	4.05 ± 0.78	4.13 ± 0.45	
POC (mg/L)	RM 8.4	4.98 ± 3.27	1.65 ± 0.84	8.95 ± 4.16	−
	RM 10.2	3.26 ± 1.52	1.16 ± 0.15	4.45 ± 1.87	
	RM 12	2.28 ± 1.13	1.88 ± 0.41	1.97 ± 0.46	
	RM 13.5	1.97 ± 0.98	1.77 ± 0.55	1.82 ± 0.55	
	RM 15.8	1.94 ± 1.41	1.49 ± 0.19	0.96 ± 0.56	
SSC (mg/L)	RM 8.4	56.46 ± 44.74	14.63 ± 8.14	85.57 ± 62.27	80–100 for fish^c^
	RM 10.2	31.66 ± 22.47	8.34 ± 1.51	36.77 ± 10.29	
	RM 12	21.05 ± 16.34	13.45 ± 4.19	12.83 ± 5.23	
	RM 13.5	11.49 ± 7.88	12.13 ± 3.12	10.87 ± 1.1	
	RM 15.8	9.4 ± 12.4	11.85 ± 1.91	1.8 ± 0.28	

^a^NJDEP (2026)

^b^ USEPA (1986)

^c^Griffiths and Walton (1978)

### Water quality indices

#### Single water quality indices

**Fig. 4:**
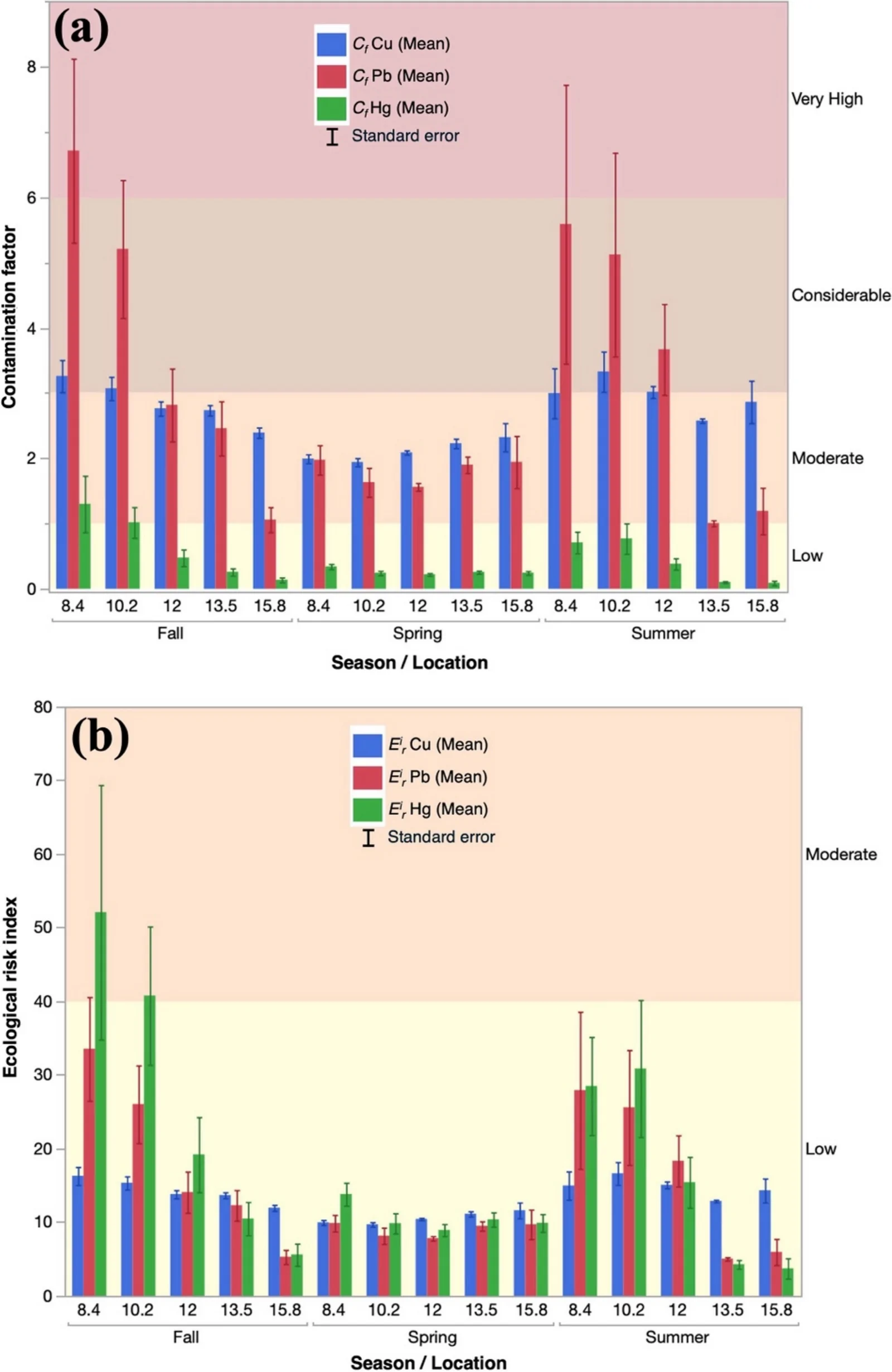
Seasonal and spatial variation of **a** contamination factor (*C*_*f*_) and **b** ecological risk index (*E*^*i*^_*r*_) for copper (Cu), lead (Pb), and mercury (Hg) across five locations (River Miles 8.4 to 15.8) along the upper portion of the Lower Passaic River (LPR). Error bars represent standard error

The contamination factor (*C*_*f*_) and ecological risk index (*E*^*i*^_*r*_) for Cu, Pb, and Hg were analyzed seasonally across five sampling locations in the LPR. The contamination factor (Fig. 4a) of Pb consistently showed higher contamination factors compared to Cu and Hg, particularly in fall and summer, with “considerable to very high” contamination levels observed at RM 8.4 and RM 10.2. In spring, Pb levels were “moderate to considerable.” Cu displayed moderate contamination levels across most seasons and locations, with the highest levels at downstream sites (e.g., RM 8.4). Hg demonstrated the lowest contamination factor among the metals, with low levels observed across most seasons and locations.

The ecological risk index (Fig. 4b) of Hg contributed significantly to the ecological risk, with moderate risks observed in fall at RM 8.4 and RM 10.2, while risks decreased to low levels in spring and summer. Pb exhibited “moderate to low” ecological risk in fall but showed a reduction to “low” risk in other seasons. Cu consistently remained in the “low” risk category across all seasons and locations. Seasonal differences highlighted fall as the period with the highest ecological risk levels for all three metals.

#### Integrated water quality indices

Integrated water quality indices (*C*_*d*_, *PERI*, *HEI*, and *HPI*) for heavy metals across five locations in the LPR during fall, spring, and summer are presented in Table 4. Values of *C*_*d*_ ranged from “low” (< 1) to “high” (> 3) across locations and seasons. The highest *C*_*d*_ values were observed at RM 8.4 and RM 10.2 during fall (11.27 and 9.296, respectively), while spring and summer showed comparatively lower values, predominantly in the “medium” to “high” range. *PERI* values were highest in fall, with RM 8.4 reaching a serious level (102) and RM 10.2 a “moderate” level (82.17). In spring and summer, *PERI* values remained in the “low” risk category (< 150). *HEI* values were low (< 400) across all locations and seasons, with the highest value observed at RM 8.4 in fall (0.168) (Table 4). *HPI* values were generally low (< 100) across all seasons and locations, with a maximum of 3.373 at RM 8.4 during fall (Table 4).

**Table 4:** Seasonal variation of integrated heavy metal pollution indices (*C*_*d*_, *PERI*, *HEI*, and *HPI*) at different locations along the Lower Passaic River (LPR)

Season	Location	Heavy metal pollution indices			
		*C*_*d*_	*PERI*	*HEI*	*HPI*
Fall	RM 8.4	11.2730	101.956	0.168	3.373
	RM 10.2	9.2960	82.167	0.129	2.481
	RM 12	6.0630	47.102	0.048	0.850
	RM 13.5	5.4590	36.487	0.052	0.914
	RM 15.8	3.5960	22.888	0.024	0.412
Spring	RM 8.4	4.3180	33.691	0.049	0.931
	RM 10.2	3.8250	27.763	0.028	0.513
	RM 12	3.8800	27.226	0.033	0.588
	RM 13.5	4.3920	31.044	0.044	0.796
	RM 15.8	4.5180	31.270	0.042	0.751
Summer	RM 8.4	9.2970	71.437	0.167	2.995
	RM 10.2	9.2200	73.122	0.045	0.829
	RM 12	7.0720	48.863	0.067	1.166
	RM 13.5	3.6950	22.229	0.022	0.365
	RM 15.8	4.1540	24.040	0.027	0.437

To provide a clear statistical justification for the observed temporal patterns, Kruskal–Wallis tests were conducted on these integrated indices. The results reveal a distinct seasonal pattern: *C*_*d*_ showed a statistically significant variation across seasons (*p* = 0.001), with a post hoc Steel–Dwass test confirming that fall values were significantly higher than spring values. In contrast, the *PERI*, along with the *HEI* and *HPI*, did not exhibit statistically significant seasonal variation (*p* > 0.05) (Table S3).

Spatially, the downstream sites, particularly RM 8.4 and RM 10.2, consistently exhibited higher contamination and ecological risk levels across indices. Table 5 shows heavy metal toxicity (Cu, Pb, and Hg) and toxicity load across locations in the LPR during fall, spring, and summer. Pb consistently exhibited the highest toxicity, peaking in summer at RM 8.4 (2.46E + 03 µg/L), while Hg shows the lowest values. The heavy metal toxicity load was highest in summer (5.49E + 03 µg/L at RM 8.4) and lowest in spring (1.91E + 03 µg/L at RM 10.2) (Table 5). However, the studied metals did not exceed their permissible toxicity load (PTL).

**Table 5:** Toxicity of heavy metals (µg/L) and heavy metal toxicity load (µg/L) in surface water of the Lower Passaic River (LPR)

Season	Location	Toxicity of heavy metals			Heavy metal toxicity load
		Cu	Pb	Hg	
Fall	RM 8.4	2.77E + 03	2.40E + 03	2.68E + 01	5.20E + 03
	RM 10.2	2.56E + 03	1.85E + 03	1.64E + 01	4.43E + 03
	RM 12	2.17E + 03	6.78E + 02	3.93E + 00	2.85E + 03
	RM 13.5	2.20E + 03	7.41E + 02	3.78E + 00	2.95E + 03
	RM 15.8	1.93E + 03	3.25E + 02	2.04E + 00	2.26E + 03
Spring	RM 8.4	1.70E + 03	6.99E + 02	5.97E + 00	2.41E + 03
	RM 10.2	1.51E + 03	3.99E + 02	2.76E + 00	1.91E + 03
	RM 12	1.71E + 03	4.61E + 02	3.06E + 00	2.17E + 03
	RM 13.5	1.76E + 03	6.29E + 02	3.93E + 00	2.40E + 03
	RM 15.8	1.88E + 03	5.96E + 02	3.61E + 00	2.48E + 03
Summer	RM 8.4	3.02E + 03	2.46E + 03	1.12E + 01	5.49E + 03
	RM 10.2	2.28E + 03	6.24E + 02	5.24E + 00	2.91E + 03
	RM 12	2.39E + 03	9.69E + 02	3.93E + 00	3.37E + 03
	RM 13.5	2.03E + 03	3.05E + 02	1.16E + 00	2.34E + 03
	RM 15.8	2.31E + 03	3.66E + 02	1.36E + 00	2.68E + 03
Hazard intensity score (HIS)^a^		807	1531	1455	−
Permissible toxicity load (PTL)		1.05E + 06	2.30E + 04	2.92E + 03	5.92E + 06

^a^ ATSDR (2025)

### Multivariate analysis

**Fig. 5:**
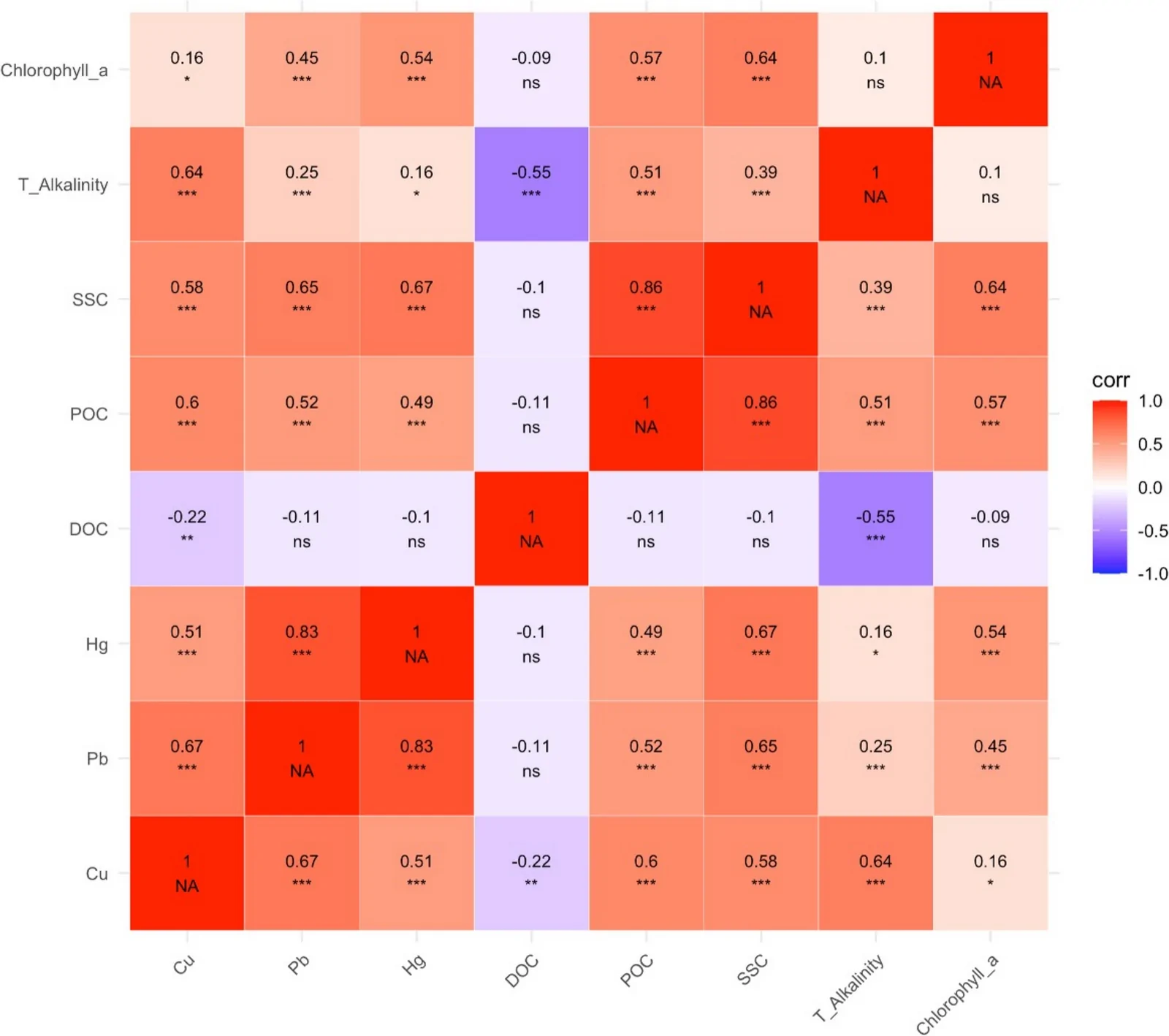
Spearman’s rank correlation matrix among heavy metals and physicochemical parameters in the Lower Passaic River

Spearman’s rank correlation analysis revealed several significant relationships among heavy metals and environmental variables (Fig. 5). The strongest association was observed between Pb and Hg (*r* = 0.83, *p* < 0.001), followed by Pb and Cu (r = 0.67, *p* < 0.001) and Cu and Hg (*r* = 0.51, *p* < 0.001), suggesting that the three metals likely share common sources or exhibit similar environmental behavior. All three metals also showed significant positive correlations with suspended sediment concentration (SSC; *r* = 0.58–0.67, *p* < 0.001) and particulate organic carbon (POC; *r* = 0.49–0.60, *p* < 0.001), indicating that particulate matter plays an important role in metal transport and accumulation. Chlorophyll-*a* was positively correlated with Pb (*r* = 0.45, *p* < 0.001) and Hg (*r* = 0.54, *p* < 0.001), whereas its relationship with Cu was weak (*r* = 0.16, *p* < 0.05), suggesting a stronger association of Pb and Hg with biological productivity.

Among the environmental variables, SSC exhibited a very strong positive correlation with POC (*r* = 0.86, *p* < 0.001), indicating that organic carbon is largely associated with suspended particulate matter. Total alkalinity was positively correlated with Cu (*r* = 0.64, *p* < 0.001), Pb (*r* = 0.25, *p* < 0.001), Hg (*r* = 0.16, *p* < 0.05), POC (*r* = 0.51, *p* < 0.001), and SSC (*r* = 0.39, *p* < 0.001), but showed a strong negative correlation with dissolved organic carbon (DOC; *r* = −0.55, *p* < 0.001). DOC was weakly or negatively correlated with most variables and was significantly negatively associated only with Cu (*r* = −0.22, *p* < 0.01), while its relationships with Pb, Hg, chlorophyll-*a*, POC, and SSC were not statistically significant. These correlations indicate that particulate matter and associated organic carbon are the dominant controls on heavy metal distribution in the study area, whereas DOC appears to play a comparatively minor role.

To detect the underlying patterns and dimensionality within the dataset, a principal component analysis (PCA) with varimax rotation was applied to physical–chemical parameters and heavy metals. The first three major components (PC1, PC2, and PC3) were retained on the basis of eigenvalue > 1 criteria, cumulatively explaining 79.56% of the total variance (Fig. S2). The PC1 was responsible for 47.04% variance and was strongly loaded with SSC (0.87), Cu (0.83), Pb (0.82), and Hg (0.66). PC2 explained 17.92% of the variance and was dominated by high positive loading for DOC (0.87) and total alkalinity (− 0.71). PC3 showed high loading accounting for 14.60% variance, chlorophyll-*a* (0.70), and Cu (0.65) (Table S4).

### Application of machine learning (ML) models for predicting PERI

**Fig. 6:**
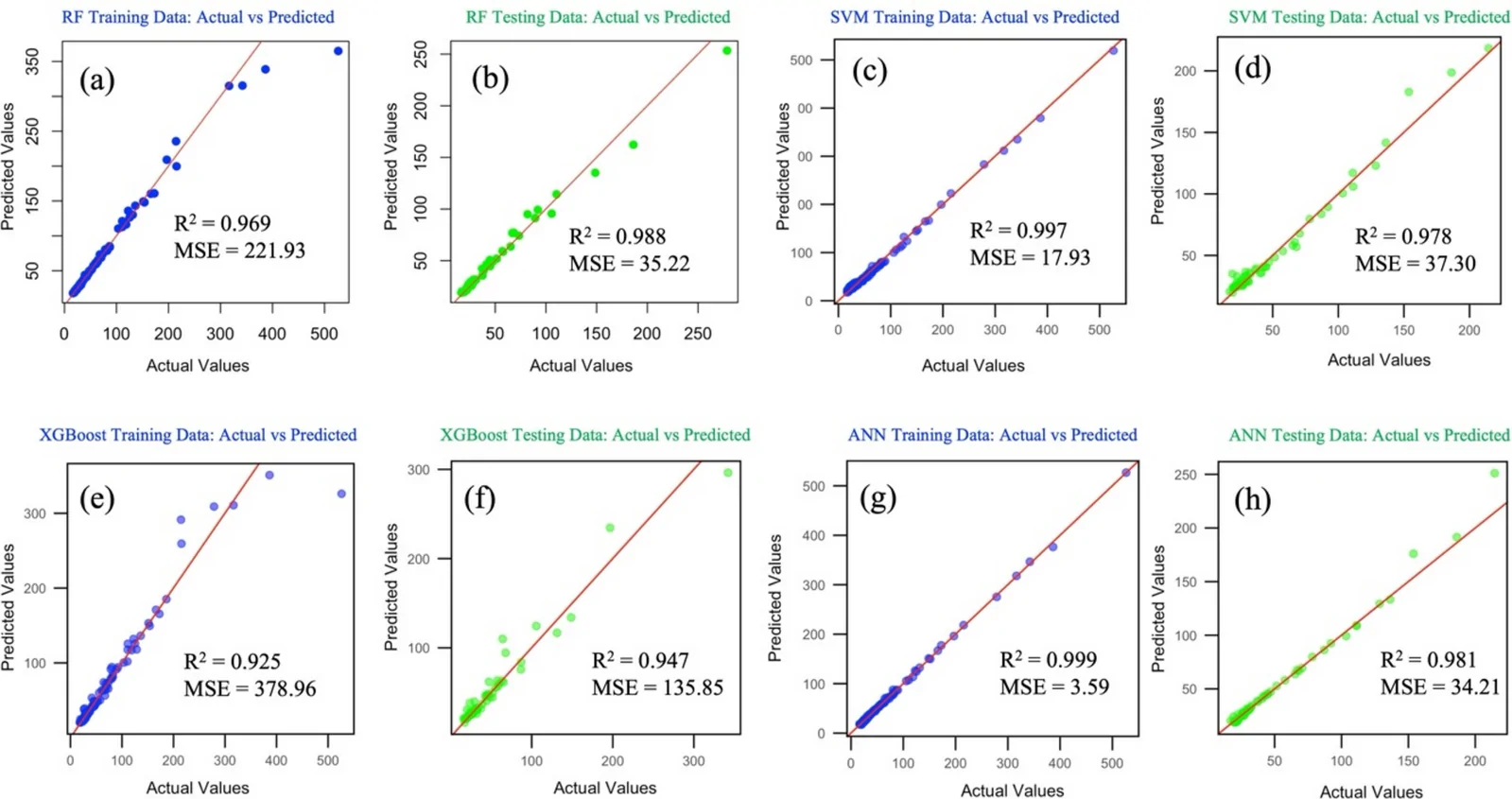
Comparison of actual and predicted *PERI* values obtained from the best-performing machine learning models: random forest (RF) (**a**, **b**), support vector machine (SVM) (**c**, **d**), extreme gradient boosting (XGBoost) (**e**, **f**), and artificial neural network (ANN) (**g**, **h**)

The performance evaluation of four machine learning (ML) models (RF, ANN, SVM, and XGBoost) for predicting the parameters *PERI* during the training and testing phases is shown in Table 6 and Fig. 6. ANN showed the strongest performance in both phases. During training, it produced the lowest MSE (3.59), MAE (1.32), and RMSE (1.90), together with the highest *R*^2^ (0.999) and NSE (0.99). Its testing results were similarly strong, with an MSE of 34.21, MAE of 2.25, RMSE of 5.85, *R*^2^ of 0.981, and NSE of 0.98. SVM ranked second, with training MSE, MAE, and RMSE of 17.93, 3.53, and 4.23, and testing values of 37.30, 4.26, and 6.11. RF delivered moderate performance, with higher training errors (MSE = 39.82, MAE = 3.08, and RMSE = 6.31) and testing errors (MSE = 28.95, MAE = 2.39, and RMSE = 5.93). XGBoost performed the weakest. Its training MSE, MAE, and RMSE were 149.08, 5.24, and 12.21, and its testing errors remained high, with an MSE of 135.85, MAE of 5.97, and RMSE of 11.66. These results confirm that ANN is the most accurate and reliable model, followed by SVM, while RF and XGBoost are less suitable for this prediction task.

**Table 6:** Performance evaluation of machine learning models (RF, SVM, XGBoost, and ANN) for predicting *PERI* during training and testing phases

	RF	SVM	XGBoost	ANN	RF	SVM	XGBoost	ANN
	Training				Testing			
MSE	39.82	17.93	149.08	3.59	28.95	37.30	135.85	34.21
MAE	3.08	3.53	5.24	1.32	2.39	4.26	5.97	2.25
RMSE	6.31	4.23	12.21	1.90	5.38	6.11	11.66	5.85
RSR	0.16	0.06	0.25	0.03	0.14	0.14	0.23	0.14
NSE	0.97	0.996	0.92	0.99	0.98	0.98	0.95	0.98
MAPE (%)	2.51	9.73	6.26	3.15	3.75	10.85	11.07	4.24
*R*^2^	0.972	0.997	0.925	0.999	0.985	0.978	0.947	0.981

### Variable importance by ML models

**Fig. 7:**
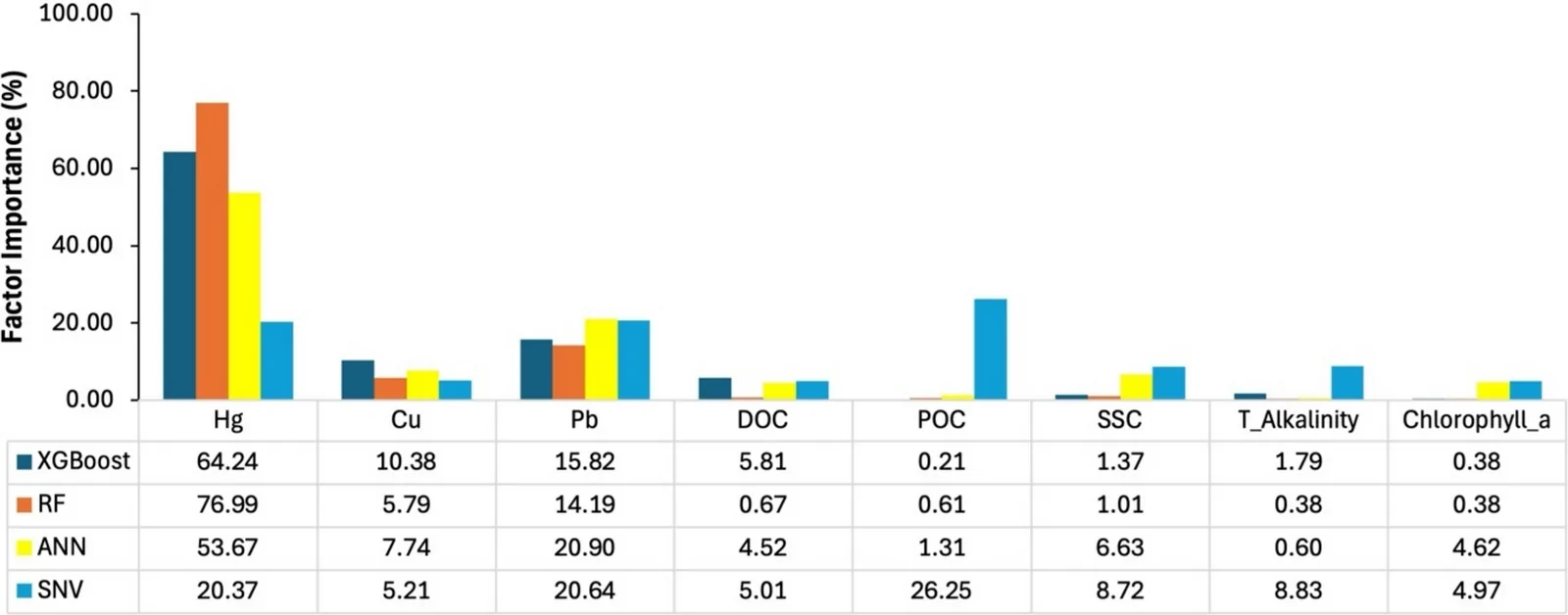
Relative importance of input parameters in predicting ecological risk index (*PERI*) using four ML models: XGBoost, RF, ANN, and SVM

The selection and significance of input variables are critical for ensuring the robustness and predictive accuracy of machine learning (ML) models. In this study, the importance of input variables in predicting the *PERI* concentration was evaluated using four ML models: XGBoost, RF, ANN, and SVM. As shown in Fig. 7, variable importance values were expressed as percentages to provide a consistent scale for comparing the relative contribution of each predictor.

Mercury emerged as the most influential predictor for *PERI*, achieving the highest relative importance across all models except SVM (XGBoost (64.24), RF (76.99), ANN (53.67), and SVM (20.37)) (Fig. 7). Conversely, Pb consistently exhibited the lowest predictive significance. Notably, Cu demonstrated greater relative importance than Pb across all four models.

## Discussion

### Interpretation of heavy metal and physicochemical dynamics

The contrasting seasonal behavior of Cu relative to Pb and Hg indicates that these metals do not share the same dominant inputs or transport mechanisms within the system (Suttie & Wolff, 1992). In this study, Cu exhibited significant seasonal variation (*p* < 0.001), with higher concentrations observed during fall and summer, particularly at RM 8.4 and RM 10.2 (Table 2 and Fig. 3). The noticeable seasonality of Cu supports the interpretation that its concentrations are strongly influenced by recent watershed inputs, particularly surface runoff that varies with precipitation and hydrological conditions (Riso et al., 1993). The elevated Cu concentrations at these downstream locations coincided with higher SSC and POC levels, suggesting that particulate matter and organic-rich materials may contribute to Cu transport and retention within the water column.

In comparison, the limited seasonal variability observed for Pb and Hg suggests that their presence in the water column is shaped primarily by long-term processes such as the slow release of legacy contaminants from bed sediments or the continuous resuspension of previously deposited material. These processes are governed less by short-term hydrological fluctuations and more by the persistent geochemical and physical characteristics of the river system, consistent with patterns reported in earlier studies (Reza & Singh, 2010; Riso et al., 1993). The relatively higher Pb and Hg concentrations observed at RM 8.4 and RM 10.2 compared with other monitoring locations further suggest that downstream areas may be influenced by historical contamination, sediment accumulation, and remobilization processes.

The seasonal variation observed in chlorophyll-*a*, especially during summer, suggests high algal productivity, likely driven by warm temperatures and increased availability of sunlight (Chen et al., 2023; Son et al., 2018). The highest chlorophyll-*a* concentrations during summer at RM 8.4 and RM 10.2 indicate enhanced biological activity at these locations, which may influence metal cycling through interactions with organic matter and biological uptake processes. The fluctuations in total alkalinity between seasons can be attributed to seasonal changes in photosynthetic activity and the dissolution of calcium carbonate, influenced by hydrological and biological factors (Ingrosso et al., 2016; Legge et al., 2017). Relatively stable DOC and POC values suggest consistent organic matter input and turnover within the system (Dordoni et al., 2022; Worrall & Moody, 2014). However, the relatively higher POC concentrations at downstream sites may contribute to localized differences in metal mobility by providing additional binding surfaces for trace metals. High SSC during summer is consistent with potentially increased surface runoff and resuspension of sediment from upstream areas due to seasonal rainfall, which can contribute to reducing water clarity and causing potential stress on aquatic ecosystems (Dugan et al., 2009; Tramblay et al., 2008). The concurrent increase in SSC and elevated Cu and Pb concentrations during summer supports the role of suspended particles as an important transport pathway for metals in the LPR.

### Interpretation of pollution indices and ecological risk

The elevated contamination factor for Pb, especially in fall and summer, reflects its historical accumulation from industrial activities and urban runoff (Donovan et al., 2008). The marked difference between the fall and spring suggests potentially seasonal input sources, such as stormwater runoff or sediment resuspension during wet periods (Sodré et al., 2012; Zhao et al., 2024). Hg, although demonstrating the lowest contamination factor, contributed significantly to ecological risk due to its high toxicity and bioavailability (Islam et al., 2025), especially in the fall at RM 8.4 and RM 10.2. This aligns with the potential for legacy contamination in sediments to mobilize under specific environmental conditions. While Pb and Cu pose moderate to low risks, Hg presents a more significant concern for aquatic ecosystems due to its high toxic response factor (Chen & Driscoll, 2018; Wiener & Suchanek, 2008). Ecological risk showed seasonal variations linked to changes in hydrological and biological factors, including sediment disturbances during high-flow periods, and can intensify during low-flow periods due to reduced dilution capacity (Bao et al., 2018; Ramos et al., 2016). These results emphasize the need for targeted mitigation strategies during high-risk seasons, particularly at downstream locations such as RM 8.4 and RM 10.2.

The high contamination degree (*C*_*d*_) observed in RM 8.4 and RM 10.2 suggests that these sites are hotspots of metal pollution (Fig. S1), which is likely influenced by legacy contamination from industrial discharge and enhanced sediment transport during certain seasons (Chen & Guestrin, 2016; Donovan et al., 2008). Lower *C*_*d*_ values in spring and summer indicate significant seasonal variations, potentially due to a dilution effect from higher water flows or reduced sediment mobilization (Sodré et al., 2012; Zhao et al., 2024). The *PERI* results highlight that while the overall ecological risk is low to moderate, RM 8.4 and RM 10.2 experience high risk in the fall due to the high bioavailability and toxicity of specific metals, such as Hg.

Spatial patterns across the LPR indicate a downstream-to-upstream gradient in contamination, with the highest pollution levels consistently observed at RM 8.4 and RM 10.2 and progressively lower values at the upstream locations (RM 12, RM 13.5, and RM 15.8) (Tables 4 and 5). The elevated contamination indices at the downstream sites likely reflect their proximity to historical industrial areas, urban drainage inputs, and zones of enhanced sediment accumulation where particle-associated metals can be retained and remobilized (Minh & Bui, 2026). In contrast, the comparatively lower contamination levels observed at upstream sites may be associated with reduced exposure to direct anthropogenic inputs, greater distance from historical discharge areas, and increased dilution and dispersion processes (Palumbo-Roe et al., 2012; Zhang et al., 2025a, b). However, the presence of measurable contamination at all locations indicates that metal transport and redistribution occur throughout the river system, likely influenced by hydrodynamic conditions, sediment dynamics, and seasonal variations in runoff (Munusamy et al., 2025; Razi & Zahratunnisa, 2026). These spatial patterns highlight the importance of considering both localized pollution sources and river-wide transport processes when evaluating ecological risks in the LPR.

Low values for both *HEI* and *HPI* indicate that the water is not heavily polluted by heavy metals and remains within the permissible range defined by regulatory bodies. Seasonal increases in toxic load, especially in summer, suggest the influence of elevated runoff. Pb dominates the toxicity burden, which indicates persistent industrial and urban sources (Wenning et al., 1994).

### Interpretation of multivariate statistical patterns

The multivariate analysis indicates that heavy metal distribution in the LPR is controlled by multiple interacting processes, including particulate transport, geochemical conditions, and biological influences. The dominance of SSC, Cu, Pb, and Hg in PC1 (Table S4), together with the significant correlations between SSC and metals (Fig. 5), suggests that suspended particulate matter is a major pathway for metal transport and redistribution. This pattern is consistent with the strong affinity of Cu, Pb, and Hg for particulate phases in aquatic systems (Feng et al., 2017; Kucuksezgin et al., 2008).

PC2, characterized by DOC and total alkalinity (Table S4), reflects the potential influence of dissolved organic matter and chemical conditions on metal mobility through processes such as complexation and changes in metal availability (Zhu et al., 2021). PC3, associated primarily with chlorophyll-*a* and Cu (Table S4), suggests that biological activity may contribute to metal cycling through interactions with algal biomass and organic matter (Devesa-Rey et al., 2009; Szymańska-Walkiewicz et al., 2022). Together, these findings demonstrate that metal behavior in the LPR results from the combined effects of sediment-associated transport, geochemical processes, and biological interactions.

### Interpretation of machine learning model performance

The model comparison showed that the artificial neural network (ANN) was the most effective approach for predicting *PERI*. It achieved the lowest errors in both training and testing, together with the highest *R*^2^ and NSE values, which indicates strong capability in capturing the nonlinear structure of the *PERI* response. Random forest (RF) produced moderate results. Its testing performance was acceptable, but its higher training errors suggest limited ability to represent the full complexity of the system. The support vector machine (SVM) ranked third. Although its performance was weaker than that of ANN, it remained stable across phases, which is consistent with the known dependence of SVM accuracy on kernel selection and parameterization (Tharwat, 2019). XGBoost showed the weakest performance, with relatively high errors in both the training and testing phases. This finding is consistent with previous studies reporting that XGBoost may not outperform other machine learning algorithms on relatively small datasets or when complex nonlinear relationships are limited, despite hyperparameter optimization (Bentéjac et al., 2021).

These findings identify ANN as the most reliable model for *PERI* prediction, followed by RF, while SVM and XGBoost were less suitable for this application. The strong performance of ANN, in particular, demonstrates the capacity of machine learning to represent the combined influence of multiple heavy metals on ecological risk. This supports a broader shift in environmental modeling toward using ML methods not only for concentration estimation but also for ecological and health risk prediction. Recent work has applied similar approaches to forecast toxicity or biological responses based on pollutant data (Bai et al., 2025; Fan et al., 2021; Jeong et al., 2024; Pantic et al., 2025; Zhang et al., 2025a, b), underscoring the growing relevance of risk-focused modeling frameworks.

The high accuracy achieved in this study (testing *R*^2^ = 0.981 and NSE = 0.98 for ANN) indicates that such models can be transferred to other urban river systems with comparable contamination profiles. Retraining the models with local input data would allow extension to additional metals or risk indices. This adaptability positions the ANN model as a useful component of rapid-assessment systems, capable of converting new concentration measurements into reliable ecological risk predictions without the need for full index recalculation. Its operational value is therefore significant for data-scarce or rapidly changing monitoring contexts.

### Interpretation of variable importance and management implications

The dominance of Hg in ecological risk prediction is likely linked to its persistent and bioaccumulative nature and to legacy industrial emissions historically discharged into the LPR (Iannuzzi & Ludwig, 2004; Olson & Tharp, 2020). This finding has critical ecological implications. Mercury’s bioaccumulation in the food web poses a threat to piscivorous (fish-eating) wildlife and human health, potentially leading to advisories on fish consumption. Although the ML algorithms showed some variation in the relative importance rankings of the predictors (Fig. 7), mercury was consistently identified as one of the most influential variables across the models. This consistent identification suggests broad agreement regarding the importance of mercury, while acknowledging algorithm-specific differences in predictor rankings. For river management, this unequivocally identifies mercury as the primary target for remediation efforts. This finding is consistent with the high toxic weighting of Hg in the *PERI* calculation, supporting the validity and robustness of the ML approach applied in this study. Therefore, any intervention strategy aimed at reducing the overall ecological risk (*PERI*) in the LPR must prioritize the control and remediation of mercury sources and their mobilization from sediments.

## Conclusion

This study provides a comprehensive analysis of heavy metal contamination (Cu, Pb, and Hg) in the surface water of the Lower Passaic River (LPR), combining traditional ecological risk indices with advanced machine learning (ML) modeling to enhance predictive accuracy and environmental insights. The findings indicate that metal concentrations and associated ecological risk display significant spatial variability, with persistent contamination and high-risk levels at downstream locations such as RM 8.4 and RM 10.2, particularly during the fall season. Among the heavy metals analyzed, Hg emerged as the most significant ecological pollutant due to its high toxic response factor, despite its relatively low concentration.

The application of ML models, including random forest (RF), support vector machine (SVM), extreme gradient boosting (XGBoost), and artificial neural network (ANN), to predict the potential ecological risk index (*PERI*) demonstrated outstanding performance. The testing results identified ANN as the top-performing model. The ANN achieved the lowest prediction errors (MAE = 2.25 and MAPE = 4.24%) and the highest coefficient of determination (*R*^2^ = 0.981), indicating excellent predictive performance and effective capture of the complex nonlinear relationships within the data. Variable importance analysis further highlighted mercury’s dominant role in driving ecological risk, corroborating the statistical findings and providing additional insight into the key predictors identified by the ML models.

This study’s key scientific contribution lies in demonstrating a paradigm shift from static to dynamic ecological risk assessment. By integrating machine learning with ecological risk indices, we have established a robust, transferable framework for quantifying, predicting, and managing heavy metal–induced ecological risks in complex urban river systems. This approach advances water quality management from reactive monitoring toward proactive, science-based decision-making by providing a mechanistic understanding of risk drivers and enabling forecasting. In practical terms, the findings provide actionable insights for the NY–NJ Harbor system, offering a transferable framework that supports pollution mitigation, restoration planning, and compliance with regional and national water quality objectives.

Based on these results, it is recommended that local water authorities integrate ML-based predictive systems into routine monitoring programs to identify contamination hotspots in real time and prioritize mitigation efforts. Continuous sampling across multiple seasons should be institutionalized to capture temporal dynamics, while stricter regulatory limits on mercury discharges should be enforced to reduce ecological risk. Collaborative monitoring between environmental agencies and research institutions is also encouraged to enhance data availability and model reliability.

This study acknowledges certain limitations. The analysis was based on a limited number of sampling campaigns and selected heavy metals (Cu, Pb, and Hg), which may not fully capture long-term temporal variations or the influence of other contaminants such as cadmium or arsenic. Additionally, while the ML models demonstrated strong predictive performance, their reliability depends on the availability and representativeness of input data. Future research should include a broader range of pollutants, incorporate hydrodynamic modeling for improved simulation of pollutant transport, and develop real-time ML-based prediction systems to enhance early warning and adaptive water quality management.

## Supplementary Information

Below is the link to the electronic supplementary material.

ESM 1 (DOCX 1.07 MB)

## Data Availability

The data utilized in this study were obtained from the Passaic River Public Digital Library (https://sharepoint.ourpassaic.org/SitePages/Passaic%20River%20Datasets.aspx).

## Abbreviations

*ANN*: Artificial neural network
*C*_*d*_: Contamination degree
*HEI*: Heavy metal evaluation index
*HPI*: Heavy metal pollution index
*LPR*: Lower Passaic River
*MAE*: Mean absolute error
*ML*: Machine learning
*NSE*: Nash–Sutcliffe efficiency
*PCA*: Principal component analysis
*PERI*: Potential ecological risk index
*R*^2^: Coefficient of determination
*RF*: Random forest
*RM*: River mile
*RMSE*: Root mean square error
*SVM*: Support vector machine
*XGBoost*: Extreme gradient boosting

## References

[CR1] Abraj, M., Wang, Y.-G., & Thompson, M. H. (2022). A new mixture copula model for spatially correlated multiple variables with an environmental application. *Scientific Reports,**12*(1), 13867. 10.1038/s41598-022-18007-z 35974067 PMC9381801

[CR2] Acharjee, M. R., Newase, S., Afrin, S., Islam, S., Hridoy, M. A. A. M., Haque, M. E., et al. (2026). From water and sediment to seafood: Heavy metal contamination and human health risk in wild and cultured oysters from a developing coastal region. *Marine Pollution Bulletin,**225*, Article 119313. 10.1016/j.marpolbul.2026.119313 41576866

[CR3] AECOM. (2022). *Small volume chemical water column monitoring – Data summary report phase 1.* Chelmsford.

[CR4] Ahmed, B., Islam, S., Quraishi, S. B., Alam, M. N. E., Ahsan, M. S., & Kabir, A. (2024). A probabilistic risk assessment of heavy metal in water and sediment: An industrially affected urban river in Bangladesh. *Water Environment Research*. 10.1002/wer.11097 39155848

[CR5] ATSDR. (2025). *Substance priority list*. https://www.atsdr.cdc.gov/programs/substance-priority-list.html?CDC_AAref_Val=https://www.atsdr.cdc.gov/spl/. Accessed 28 Apr 2026.

[CR6] Bai, C., Wu, L., Li, R., Cao, Y., He, S., & Bo, X. (2025). Machine learning‐enabled drug‐induced toxicity prediction. *Advanced Science*. 10.1002/advs.202413405 PMC12021114 39899688

[CR7] Bao, K., Liu, J., You, X., Shi, X., & Meng, B. (2018). A new comprehensive ecological risk index for risk assessment on Luanhe River, China. *Environmental Geochemistry and Health,**40*(5), 1965–1978. 10.1007/s10653-017-9978-6 28573332

[CR8] Basirian, S., Najafzadeh, M., & Demir, I. (2026). Water quality monitoring for coastal hypoxia: Integration of satellite imagery and machine learning models. *Marine Pollution Bulletin, 222*, 118735. 10.1016/j.marpolbul.2025.118735 41037951

[CR9] Bentéjac, C., Csörgő, A., & Martínez-Muñoz, G. (2021). A comparative analysis of gradient boosting algorithms. *Artificial Intelligence Review,**54*(3), 1937–1967. 10.1007/s10462-020-09896-5

[CR10] Bi, Z., Sun, J., Xie, Y., Gu, Y., Zhang, H., Zheng, B., et al. (2024). Machine learning-driven source identification and ecological risk prediction of heavy metal pollution in cultivated soils. *Journal of Hazardous Materials,**476*, Article 135109. 10.1016/j.jhazmat.2024.135109 38972204

[CR11] Breiman, L. (2001). Random Forests. *Machine Learning, 45*(1), 5–32. 10.1023/A:1010933404324

[CR12] Chakraborty, T. K., Ghosh, G. C., Ghosh, P., Jahan, I., Zaman, S., Islam, M. S., et al. (2022). Arsenic, iron, and manganese in groundwater and its associated human health risk assessment in the rural area of Jashore, Bangladesh. *Journal of Water and Health, 20*(6), 888–902. 10.2166/wh.2022.284 35768965

[CR13] Chakraborty, T. K., Islam, M. S., Ghosh, G. C., Ghosh, P., Zaman, S., Habib, A., et al. (2023). Human health risk and hydro-geochemical appraisal of groundwater in the southwest part of Bangladesh using GIS, water quality indices, and multivariate statistical approaches. *Toxin Reviews,**42*(1), 285–299. 10.1080/15569543.2022.2134572

[CR14] Chen, C. Y., & Driscoll, C. T. (2018). Integrating mercury research and policy in a changing world. *Ambio,**47*(2), 111–115. 10.1007/s13280-017-1010-y 29388125 PMC5794685

[CR15] Chen, T., & Guestrin, C. (2016). XGBoost. In *Proceedings of the 22nd ACM SIGKDD International Conference on Knowledge Discovery and Data Mining* (pp. 785–794). ACM. 10.1145/2939672.2939785

[CR16] Chen, Y., Zhao, H., & Shen, C. (2023). Summer Chlorophyll-a Increase Induced by Upwelling off the Northeastern Coast of Hainan Island, South China Sea. *Water, 15*(15), 2770. 10.3390/w15152770

[CR17] Cortes, C., & Vapnik, V. (1996). Support-vector networks. *Machine Learning, 20*, 273–297.

[CR18] de Myttenaere, A., Golden, B., Le Grand, B., & Rossi, F. (2016). Mean Absolute Percentage Error for regression models. *Neurocomputing, 192*, 38–48. 10.1016/j.neucom.2015.12.114

[CR19] Devesa-Rey, R., Moldes, A. B., Díaz-Fierros, F., & Barral, M. T. (2009). Study of phytopigments in river bed sediments: Effects of the organic matter, nutrients and metal composition. *Environmental Monitoring and Assessment,**153*(1–4), 147–159. 10.1007/s10661-008-0345-z 18597180

[CR20] Donovan, E., Unice, K., Roberts, J. D., Harris, M., & Finley, B. (2008). Risk of Gastrointestinal Disease Associated with Exposure to Pathogens in the Water of the Lower Passaic River. *Applied and Environmental Microbiology, 74*(4), 994–1003. 10.1128/AEM.00601-07 18156342 PMC2258582

[CR21] Dordoni, M., Seewald, M., Rinke, K., Friese, K., van Geldern, R., Schmidmeier, J., & Barth, J. A. C. (2022). Mineralization of autochthonous particulate organic carbon is a fast channel of organic matter turnover in Germany’s largest drinking water reservoir. *Biogeosciences, 19*(22), 5343–5355. 10.5194/bg-19-5343-2022

[CR22] Dugan, H. A., Lamoureux, S. F., Lafrenière, M. J., & Lewis, T. (2009). Hydrological and sediment yield response to summer rainfall in a small high Arctic watershed. *Hydrological Processes, 23*(10), 1514–1526. 10.1002/hyp.7285

[CR23] Essamlali, I., Nhaila, H., & El Khaili, M. (2024). Advances in machine learning and IoT for water quality monitoring: A comprehensive review. *Heliyon, 10*(6), e27920. 10.1016/j.heliyon.2024.e27920 38533055 PMC10963334

[CR24] Fan, J., Huang, G., Chi, M., Shi, Y., Jiang, J., Feng, C., et al. (2021). Prediction of chemical reproductive toxicity to aquatic species using a machine learning model: An application in an ecological risk assessment of the Yangtze River, China. *Science of the Total Environment,**796*, Article 148901. 10.1016/j.scitotenv.2021.148901 34265613

[CR25] Feng, C., Guo, X., Yin, S., Tian, C., Li, Y., & Shen, Z. (2017). Heavy metal partitioning of suspended particulate matter–water and sediment–water in the Yangtze Estuary. *Chemosphere, 185*, 717–725. 10.1016/j.chemosphere.2017.07.075 28732332

[CR26] Griffiths, W. H., & Walton, B. D. (1978). *The effects of sedimentation on the aquatic biota*. https://publications.gc.ca/site/eng/9.946490/publication.html. Accessed 28 Apr 2026.

[CR27] Hakanson, L. (1980). An ecological risk index for aquatic pollution control.a sedimentological approach. *Water Research, 14*(8), 975–1001. 10.1016/0043-1354(80)90143-8

[CR28] Hu, T., Liu, Y., Xiao, H., Pan, H., Gao, X., & Chen, Z. (2026). Quantifying drivers of rural drinking water quality using interpretable machine learning. *Environmental Impact Assessment Review, 120*, 108427. 10.1016/j.eiar.2026.108427

[CR29] Iannuzzi, T.J., & Ludwig, D. F. (2004). Historical and current ecology of the lower Passaic River. *Urban Habitats, 2*, 147–173.

[CR30] Iannuzzi, T. J., Armstrong, T. N., Long, E. R., Iannuzzi, J., & Ludwig, D. F. (2008). Sediment quality triad assessment of an industrialized estuary of the northeastern USA. *Environmental Monitoring and Assessment,**139*(1–3), 257–275. 10.1007/s10661-007-9832-x 17588158

[CR31] Ingrosso, G., Giani, M., Comici, C., Kralj, M., Piacentino, S., De Vittor, C., & Del Negro, P. (2016). Drivers of the carbonate system seasonal variations in a Mediterranean gulf. *Estuarine, Coastal and Shelf Science, 168*, 58–70. 10.1016/j.ecss.2015.11.001

[CR32] Islam, M. S., Mirza, S., Feng, H., Chakraborty, T. K., Qian, Y., & Yoo, S. (2025). Source apportionment and potential health risks of trace metals in a contaminated urban river in New York/New Jersey Harbor System. *Water,**17*(22), Article 3254. 10.3390/w17223254

[CR33] Islam, M. S., Aseperi, J., & Feng, H. (2026). Distribution, risk assessment, and source identification of metal(loid)s in sediments on a New York-New Jersey harbor superfund site. *Science of the Total Environment,**1048*, Article 182032. 10.1016/j.scitotenv.2026.182032 42520659

[CR34] Jeong, H., Park, S., Choi, B., Yu, C. S., Hong, J. Y., Jeong, T.-Y., & Cho, K. H. (2024). Machine learning-based water quality prediction using octennial in-situ Daphnia magna biological early warning system data. *Journal of Hazardous Materials, 465*, 133196. 10.1016/j.jhazmat.2023.133196 38141299

[CR35] Khairy, M., Barrett, K., & Lohmann, R. (2015). Changing sources of polychlorinated dibenzo-*p*-dioxins and furans in sediments and ecological risk for nekton in the lower Passaic River and Newark Bay, New Jersey, USA. *Environmental Toxicology and Chemistry, 35*(3), 550–562. 10.1002/etc.3223 26315691

[CR36] Kucuksezgin, F., Uluturhan, E., & Batki, H. (2008). Distribution of heavy metals in water, particulate matter and sediments of Gediz River (Eastern Aegean). *Environmental Monitoring and Assessment,**141*(1–3), 213–225. 10.1007/s10661-007-9889-6 17846908

[CR37] Laarne, P., Amnell, E., Zaidan, M. A., Mikkonen, S., & Nieminen, T. (2022). Exploring non-linear dependencies in atmospheric data with mutual information. *Atmosphere,**13*(7), Article 1046. 10.3390/atmos13071046

[CR38] Lee, J.-W., Choi, H., Hwang, U.-K., Kang, J.-C., Kang, Y. J., Kim, K. Il, & Kim, J.-H. (2019). Toxic effects of lead exposure on bioaccumulation, oxidative stress, neurotoxicity, and immune responses in fish: A review. *Environmental Toxicology and Pharmacology, 68*, 101–108. 10.1016/j.etap.2019.03.010 30884452

[CR39] Legge, O. J., Bakker, D. C. E., Meredith, M. P., Venables, H. J., Brown, P. J., Jones, E. M., & Johnson, M. T. (2017). The seasonal cycle of carbonate system processes in Ryder Bay, West Antarctic Peninsula. *Deep Sea Research Part II: Topical Studies in Oceanography, 139*, 167–180. 10.1016/j.dsr2.2016.11.006

[CR40] Li, J., Yang, S., Wang, F., Gao, M., He, L., Zhao, G., et al. (2024). Ecological risk assessment of heavy metal(loid)s in riverine sediments along the East China Sea: A large-scale integrated analysis. *Marine Pollution Bulletin,**203*, Article 116382. 10.1016/j.marpolbul.2024.116382 38678739

[CR41] Liang, Y., Ding, F., Liu, L., Yin, F., Hao, M., Kang, T., et al. (2025). Monitoring water quality parameters in urban rivers using multi-source data and machine learning approach. *Journal of Hydrology,**648*, Article 132394. 10.1016/j.jhydrol.2024.132394

[CR42] Mansor, M., Fatehah, M. O., Aziz, H. A., & Wang, L. K. (2023). Occurrence, Behaviour and Transport of Heavy Metals from Industries in River Catchments (pp. 205–277). 10.1007/978-3-031-46747-9_6

[CR43] Minh, T. T., & Bui, D. N. (2026). Co-contamination of heavy metals and PFAS in surface waters across a river estuary coast continuum under monsoonal seasonality. *Journal of Coastal Conservation,**30*(3), 37. 10.1007/s11852-026-01221-6

[CR44] Mohan, S., Kumar, B., & Nejadhashemi, A. P. (2025). Integration of machine learning and remote sensing for water quality monitoring and prediction: A review. *Sustainability,**17*(3), 998. 10.3390/su17030998

[CR45] Munusamy, C., Bhaskaran, J., Ravindran, L. A., Kolandhasamy, P., Partheeban, E. C., & Rajendran, R. (2025). Ecological health risk assessment of heavy metals in sediments and freshwater fishes: Tropical Cauvery river, South India. *Environmental Earth Sciences,**84*(16), Article 489. 10.1007/s12665-025-12492-x

[CR46] NJDEP. (2026). *Surface Water Quality Standards*. Trenton. https://dep.nj.gov/wms/bears/surface-water-quality-standards-swqs/. Accessed 28 Apr 2026.

[CR47] NOAA. (2007). *Diamond Alkali Superfund Site*. https://www.gc.noaa.gov/gc-rp/passaic_nrda_APdraft110907.pdf. Accessed 2 Feb 2026.

[CR48] Nour, H. E., & Garoub, M. M. (2026). Heavy metal bioaccumulation and risk assessment in intertidal molluscan shells and sediments of Suez Bay. *Marine Pollution Bulletin,**224*, Article 119141. 10.1016/j.marpolbul.2025.119141 41411944

[CR49] Olson, K. R., & Tharp, M. (2020). How did the Passaic River, a Superfund site near Newark, New Jersey, become an Agent Orange dioxin TCDD hotspot? *Journal of Soil and Water Conservation*, *75*(2). 10.2489/jswc.75.2.33A

[CR50] Palumbo-Roe, B., Wragg, J., & Banks, V. J. (2012). Lead mobilisation in the hyporheic zone and river bank sediments of a contaminated stream: Contribution to diffuse pollution. *Journal of Soils and Sediments,**12*(10), 1633–1640. 10.1007/s11368-012-0552-7

[CR51] Pandey, S., & Kumari, N. (2023). Impact assessment of heavy metal pollution in surface water bodies. *Metals in water* (pp. 129–154). Elsevier. 10.1016/B978-0-323-95919-3.00004-5

[CR52] Pantic, I. V., Paunovic Pantic, J., Valjarevic, S., Corridon, P. R., & Topalovic, N. (2025). Artificial intelligence – based approaches based on random forest algorithm for signal analysis: Potential applications in detection of chemico - biological interactions. *Chemico-Biological Interactions,**418*, Article 111624. 10.1016/j.cbi.2025.111624 40582691

[CR53] Proshad, R., Islam, S., Tusher, T. R., Zhang, D., Khadka, S., Gao, J., & Kundu, S. (2021). Appraisal of heavy metal toxicity in surface water with human health risk by a novel approach: A study on an urban river in vicinity to industrial areas of Bangladesh. *Toxin Reviews,**40*(4), 803–819. 10.1080/15569543.2020.1780615

[CR54] Ramos, V., Maia, R., Formigo, N., & Oliveira, B. (2016). Assessment of ecological risk based on projected hydrological alteration. *Environmental Processes,**3*(3), 569–587. 10.1007/s40710-016-0164-0

[CR55] Razi, M. H., & Zahratunnisa. (2026). Spatiotemporal dynamics of potentially toxic elements (PTEs) in the North Jakarta Estuary under anthropogenic alteration: Geochemical signatures, pollution severity, and ecological risks. *Marine Pollution Bulletin*, *227*, 119427. 10.1016/j.marpolbul.2026.119427 41713320

[CR56] Reza, R., & Singh, G. (2010). Heavy metal contamination and its indexing approach for river water. *International Journal of Environmental Science and TechnoloGy,**7*(4), 785–792. 10.1007/BF03326187

[CR57] Riso, R. D., le Corre, P., Madec, C., Birrien, J. L., & Quentel, F. (1993). Seasonal variation of copper, nickel and lead in western Brittany coastal waters (France). *Estuarine, Coastal and Shelf Science,**37*(3), 313–327. 10.1006/ecss.1993.1059

[CR58] Rumelhart, D. E., Hinton, G. E., & Williams, R. J. (1986). Learning representations by back-propagating errors. *Nature,**323*(6088), 533–536. 10.1038/323533a0

[CR59] Saha, P., & Paul, B. (2019). Assessment of heavy metal toxicity related with human health risk in the surface water of an industrialized area by a novel technique. *Human and Ecological Risk Assessment: An International Journal,**25*(4), 966–987. 10.1080/10807039.2018.1458595

[CR60] Salomons, W., & Förstner, U. (1984). Metals in continental water. *Metals in the hydrocycle* (pp. 138–211). Springer Berlin Heidelberg. 10.1007/978-3-642-69325-0_5

[CR61] Sodré, F. F., Schnitzler, D. C., Scheffer, E. W. O., & Grassi, M. T. (2012). Evaluating copper behavior in urban surface waters under anthropic influence. A case study from the Iguaçu River, Brazil. *Aquatic Geochemistry,**18*(5), 389–405. 10.1007/s10498-012-9162-7

[CR62] Son, Y.-T., Park, J.-H., & Nam, S. (2018). Summertime episodic chlorophyll *a* blooms near the east coast of the Korean Peninsula. *Biogeosciences,**15*(16), 5237–5247. 10.5194/bg-15-5237-2018

[CR63] Suttie, E. D., & Wolff, E. W. (1992). Seasonal input of heavy metals to Antarctic snow. *Tellus b: Chemical and Physical Meteorology,**44*(4), 351. 10.3402/tellusb.v44i4.15462

[CR64] Szymańska-Walkiewicz, M., Glińska-Lewczuk, K., Burandt, P., & Obolewski, K. (2022). Phytoplankton sensitivity to heavy metals in Baltic coastal lakes. *International Journal of Environmental Research and Public Health,**19*(7), Article 4131. 10.3390/ijerph19074131 35409822 PMC8998715

[CR65] Tharwat, A. (2019). Parameter investigation of support vector machine classifier with kernel functions. *Knowledge and Information Systems,**61*(3), 1269–1302. 10.1007/s10115-019-01335-4

[CR66] Tramblay, Y., St-Hilaire, A., & Ouarda, T. B. M. J. (2008). Frequency analysis of maximum annual suspended sediment concentrations in North America / Analyse fréquentielle des maximums annuels de concentration en sédiments en suspension en Amérique du Nord. *Hydrological Sciences Journal,**53*(1), 236–252. 10.1623/hysj.53.1.236

[CR67] USEPA. (1986). *National Recommended Water Quality Criteria - Aquatic Life Criteria Table*. Washington, D.C. https://www.epa.gov/wqc/national-recommended-water-quality-criteria-aquatic-life-criteria-table. Accessed 28 Apr 2026.

[CR68] USEPA. (1996). *Sampling Ambient Water for Trace Metals at EPA Water Quality Criteria Levels*. https://www.epa.gov/sites/default/files/2015-10/documents/method_1669_1996.pdf. Accessed 28 Apr 2026.

[CR69] USEPA. (1999). *Screening Level Ecological Risk Assessment Protocol for Hazardous Waste Combustion Facilities. Appendix E: Toxicity Reference Values*. Washington, DC, USA. https://archive.epa.gov/epawaste/hazard/tsd/td/web/pdf/appx-e.pdf. Accessed 28 Feb 2025.

[CR70] USEPA. (2023). *Interim EPA Cleanup Plan for the Lower Passaic River Study Area of the Diamond Alkali Superfund Site Will Remove Sources of Contamination from the River*. https://www.epa.gov/newsreleases/interim-epa-cleanup-plan-lower-passaic-river-study-area-diamond-alkali-superfund-site. Accessed 28 Feb 2025.

[CR71] USEPA. (2024). *National Primary Drinking Water Regulations*. https://www.epa.gov/ground-water-and-drinking-water/national-primary-drinking-water-regulations. Accessed 28 Apr 2026.

[CR72] USEPA. (2026). *Superfund Sites in Reuse in New Jersey*. Washington. https://www.epa.gov/superfund-redevelopment/superfund-sites-reuse-new-jersey. Accessed 6 May 2026.

[CR73] Vapnik, V. N. (1999). An overview of statistical learning theory. *IEEE Transactions on Neural Networks,**10*(5), 988–999. 10.1109/72.788640 18252602

[CR74] Wallin, J. M., Hattersley, M. D., Ludwig, D. F., & Iannuzzi, T. J. (2002). Historical assessment of the impacts of chemical contaminants in sediments on benthic invertebrates in the tidal Passaic River, New Jersey. *Human and Ecological Risk Assessment: An International Journal,**8*(5), 1155–1176. 10.1080/1080-700291905864

[CR75] Wang, H., Yilihamu, Q., Yuan, M., Bai, H., Xu, H., & Wu, J. (2020). Prediction models of soil heavy metal(loid)s concentration for agricultural land in Dongli: A comparison of regression and random forest. *Ecological Indicators,**119*, Article 106801. 10.1016/j.ecolind.2020.106801

[CR76] Wang, Y., Zhao, Y., & Xu, S. (2022). Application of VNIR and machine learning technologies to predict heavy metals in soil and pollution indices in mining areas. *Journal of Soils and Sediments,**22*(10), 2777–2791. 10.1007/s11368-022-03263-3

[CR77] Wenning, R. J., Bonnevie, N. L., & Huntley, S. L. (1994). Accumulation of metals, polychlorinated biphenyls, and polycyclic aromatic hydrocarbons in sediments from the lower Passaic River, New Jersey. *Archives of Environmental Contamination and Toxicology*, *27*(1). 10.1007/BF00203890

[CR78] Wiener, J. G., & Suchanek, T. H. (2008). The basis for ecotoxicological concern in aquatic ecosystems contaminated by historical mercury mining. *Ecological Applications*, *18*(sp8). 10.1890/06-1939.1 19475915

[CR79] Worrall, F., & Moody, C. S. (2014). Modeling the rate of turnover of DOC and particulate organic carbon in a UK, peat-hosted stream: Including diurnal cycling in short-residence time systems. *Journal of Geophysical Research: Biogeosciences,**119*(10), 1934–1946. 10.1002/2014JG002671

[CR80] Xie, Z., Liu, W., Chen, S., Yao, R., Yang, C., Zhang, X., et al. (2025). Machine learning approaches to identify hydrochemical processes and predict drinking water quality for groundwater environment in a metropolis. *Journal of Hydrology: Regional Studies,**58*, Article 102227. 10.1016/j.ejrh.2025.102227

[CR81] Yan, Y., & Yang, Y. (2025). Revealing the synergistic spatial effects in soil heavy metal pollution with explainable machine learning models. *Journal of Hazardous Materials,**482*, Article 136578. 10.1016/j.jhazmat.2024.136578 39577285

[CR82] Zhang, J., Li, X., Chen, H., Liu, G., Yan, X., & Yan, B. (2025a). Unraveling the ecotoxicity of micro(nano)plastics loaded with environmental pollutants using ensemble machine learning. *Journal of Hazardous Materials,**495*, Article 138911. 10.1016/j.jhazmat.2025.138911 40516463

[CR83] Zhang, X., Bai, M., Dong, A., Du, X., Ding, Y., & Zhao, K. (2025b). Contribution to distribution and toxicity prediction of organic pollutants in receiving waters from wastewater plant tailwater: A case study of the Yitong River, China. *Water,**17*(14), Article 2061. 10.3390/w17142061

[CR84] Zhao, J., Xu, Z., Wang, X., Wan, S., Chen, W., Huang, W., et al. (2024). Environmental copper exposure, placental cuproptosis, and miscarriage. *Environmental Pollution,**348*, Article 123847. 10.1016/j.envpol.2024.123847 38552771

[CR85] Zhu, K., Hopwood, M. J., Groenenberg, J. E., Engel, A., Achterberg, E. P., & Gledhill, M. (2021). Influence of pH and dissolved organic matter on iron speciation and apparent iron solubility in the Peruvian shelf and slope region. *Environmental Science & Technology,**55*(13), 9372–9383. 10.1021/acs.est.1c02477 34110803

[CR86] Zhu, M., Wang, J., Yang, X., Zhang, Y., Zhang, L., Ren, H., et al. (2022). A review of the application of machine learning in water quality evaluation. *Eco-Environment & Health,**1*(2), 107–116. 10.1016/j.eehl.2022.06.001 38075524 PMC10702893

