# Supplementary material for: Predictive modeling of heavy metal pollution and ecological risk for sustainable water quality management in the NY–NJ harbor system
Source: Environ Monit Assess. 2026 Aug 31;198(9):1013. doi: 10.1007/s10661-026-15832-x (PMC13529837; doi:10.1007/s10661-026-15832-x)
Supplement: Supplementary file 1 [file 10661_2026_15832_MOESM1_ESM.docx]

**Supplementary Materials**

**Table S1** Summary of the detection frequency and reporting limits (RLs) for heavy metals in water samples collected from Lower Passaic River RM 8.3 to Dundee Dam

| Trace metal | Detection frequency | RL or Range of RLs (µg/L) |
| --- | --- | --- |
| Cu | 100% | na |
| Pb | 100% | na |
| Hg | 100% | na |

*na = not applicable

**Table S2** Detailed information about machine learning models

| **Model** | **Indices** | **Mathematical Expression** | **Description** | **References** |
| --- | --- | --- | --- | --- |
| **Extreme gradient boosting (XGBoost)** | Prediction output | $ŷ_{i}=\sum_{k=1}^{K} f_{k}(x_{i}), f_{k}\in F$  Objective function: $Obj\left( \theta\right)=\sum_{i=1}^{n} l\left( y_{i},\hat{y}_{i} \right)+\sum_{k=1}^{K} \Omega\left( f_{x} \right)$  Regularization term: $\Omega(f_{k})=\gamma T+\frac{1}{2}\lambda\left\Vert\omega\right\Vert^{2}$ | Where, $ŷ$= predicted output for the 𝑖^𝑡ℎ^ instance,  $f_{k}(x_{i})$= independent regression tree (weak learner),  F = ensemble of decision trees,  $l\left( y_{i},\hat{y}_{i} \right)$= differentiable convex loss function (measuring prediction error),  $\Omega\left( f_{x} \right)$= regularization function to prevent overfitting,  $\gamma$= tree complexity penalty term,  *T* = number of terminal nodes (leaves) in each tree,  $\lambda$= regularization parameter controlling the L2-regularization on leaf weights, and  $\omega$= vector of leaf weights. | Chen and Guestrin (2016) |
| **Artificial Neural Network (ANN)** | Neuron produced output | $outputs, A=f(\sum_{q=0}^{n} u_{qp}v_{q})$ | Where, f = non-linear function,  u_q_ = q^th^ input to the neuron,  v_q_ = q^th^ synaptic weight, and  n = the number of inputs  Where, X = input value,  NX = standardized value,  p = number of patterns, and  q = value of variables | Gómez-Sanchis et al. (2006) |
|  | Normalization | $NX= \frac{X_{(p,q)}-min(q)}{\max\left( q \right)-min(q)}$ |  |  |
| **Support Vector Machine (SVM)** | SVM operated formula | $\hat{S_{0}}= \sum_{p=1}^{n} K\left( R_{p},R_{0} \right)\left( \beta_{p}-\beta_{p}^{*} \right);K\left( R_{p},R_{0} \right)$  = exp ${(-\left\vert R_{p}-R_{q} \right\vert)}^{2}/S$ | Where, $\beta_{p}$ and $\beta_{p}^{*}$ = support vectors,  K (X_i_, X_0_) = radial basis kernel function, | Singh et al. (2011) |
| **Random forest (RF)** |  | RF, every tree could be trained the other trees at the same time indicating the equal importance of all trees. The built-in feature of RF helps to measure the model structures without escaping the other parameters, and the "bagging" process assists in avoiding the resemblance between trees in the forest and keeping their diversity. On the other hand, RF can handle discrete and continuous data and produce better predictions without regulating the data set. The applied RF model has three factors including tree number (n), terminal trees (m), and leaf size (z), and selected as 500, 3, and 5, input data, respectively. |  | Fang et al. (2024) |

**Table S3** Kruskal–Wallis and Steel–Dwass test results for seasonal variation in heavy metal pollution indices (*C_d_*, *PERI*, *HEI*, *HPI*)

| Pollution indices | Steel-Dwass Test | | | Kruskal-Wallis Test | | |
| --- | --- | --- | --- | --- | --- | --- |
|  | Fall | Spring | Summer | ChiSquare | DF | p |
| *C_d_* | A | B | A | 15.36 | 2 | **0.0005** |
| *PERI* | C | C | C | 5.38 | 2 | 0.0677 |
| *HPI* | D | D | D | 3.38 | 2 | 0.1850 |
| *HEI* | E | E | E | 3.74 | 2 | 0.1539 |

* Levels not connected by the same letter are significantly different

**Table S4** Principal component analysis (PCA) with varimax rotation of physicochemical parameters and heavy metals in surface water of Lower Passaic River.

| Component | Initial Eigenvalues | | | Variables | Loading Matrix | | |
| --- | --- | --- | --- | --- | --- | --- | --- |
|  | Total | % of Variance | Cumulative % |  | PC1 | PC2 | PC3 |
| 1 | 3.76 | 47.04 | 47.04 | Cu | 0.83 | 0.12 | -0.37 |
| 2 | 1.43 | 17.92 | 64.96 | Pb | 0.82 | 0.21 | -0.44 |
| 3 | 1.17 | 14.60 | 79.56 | Hg | 0.66 | -0.09 | -0.35 |
| 4 | 0.63 | 7.90 | 87.46 | Chlorophyll-*a* | 0.45 | 0.003 | 0.70 |
| 5 | 0.57 | 7.12 | 94.58 | T Alkalinity [As CaCO_3_] | 0.51 | -0.71 | 0.14 |
| 6 | 0.34 | 4.19 | 98.77 | DOC | -0.29 | 0.87 | 0.06 |
| 7 | 0.06 | 0.76 | 99.53 | POC | 0.82 | 0.24 | 0.39 |
| 8 | 0.04 | 0.47 | 100 | SSC | 0.87 | 0.24 | 0.23 |
| Variables | Partial Contribution of Variables | | | Variables | Eigenvectors | | |
|  | PC1 | PC2 | PC3 |  | PC1 | PC2 | PC3 |
| Cu | 18.43 | 1.02 | 11.47 | Cu | 0.43 | 0.002 | 0.65 |
| Pb | 17.70 | 3.21 | 16.33 | Pb | 0.42 | -0.59 | 0.13 |
| Hg | 11.66 | 0.57 | 10.17 | Hg | 0.34 | 0.73 | 0.06 |
| Chlorophyll-*a* | 5.40 | 0.00086 | 42.34 | Chlorophyll-*a* | 0.23 | 0.003 | 0.65 |
| T Alkalinity [As CaCO_3_] | 6.98 | 34.80 | 1.56 | T Alkalinity [As CaCO_3_] | 0.26 | -0.59 | 0.13 |
| DOC | 2.26 | 52.60 | 0.31 | DOC | -0.15 | 0.73 | 0.06 |
| POC | 17.62 | 3.88 | 13.24 | POC | 0.42 | 0.20 | 0.36 |
| SSC | 19.97 | 3.92 | 4.58 | SSC | 0.45 | 0.20 | 0.21 |

**Fig. S1** Seasonal spatial distribution of contamination degree (*C_d_*) in the Lower Passaic River during (a) fall, (b) summer, and (c) spring

**Fig. S2** Scree plot representing the eigenvalues

Chen, T., & Guestrin, C. (2016). *XGBoost: A Scalable Tree Boosting System* Proceedings of the 22nd ACM SIGKDD International Conference on Knowledge Discovery and Data Mining, San Francisco, California, USA. <https://doi.org/10.1145/2939672.2939785>

Fang, W., Ren, K., Liu, T., Shang, J., Jia, S., Jiang, X., & Zhang, J. (2024). An evaluation of random forest based input variable selection methods for one month ahead streamflow forecasting. *Scientific Reports*, *14*(1), 29766. <https://doi.org/10.1038/s41598-024-81502-y>

Gómez-Sanchis, J., Martín-Guerrero, J. D., Soria-Olivas, E., Vila-Francés, J., Carrasco, J. L., & del Valle-Tascón, S. (2006). Neural networks for analysing the relevance of input variables in the prediction of tropospheric ozone concentration. *Atmospheric Environment*, *40*(32), 6173-6180. <https://doi.org/https://doi.org/10.1016/j.atmosenv.2006.04.067>

Singh, K. P., Basant, N., & Gupta, S. (2011). Support vector machines in water quality management. *Analytica Chimica Acta*, *703*(2), 152-162. <https://doi.org/https://doi.org/10.1016/j.aca.2011.07.027>

**Reference**
